# Earth pressure analysis of inclined support structures adjacent to existing structures

**DOI:** 10.1371/journal.pone.0354972

**Published:** 2026-07-30

**Authors:** Chucai Peng, Xuhui Fu, Weiwei Wang, Rui Yin

**Affiliations:** Hunan Institute of Science and Technology, Yueyang, China; Henan Polytechnic University, CHINA

## Abstract

This study investigates the earth pressure acting on inclined support structures adjacent to existing structures by combining laboratory physical model tests with theoretical derivation. Five groups of model tests were conducted at a constant width-to-height ratio of 0.95, with support inclination angles of 0°, 5°, 10°, 15°, and 20°. Particle image velocimetry (PIV) and multi-point earth pressure monitoring were employed to reveal the failure surface development in the finite soil mass and the distribution characteristics of earth pressure behind the wall. Based on the assumptions of straight failure surfaces and circular-arc major principal stress trajectories, the finite soil mass was divided into several zones. Force equilibrium equations were established for horizontal differential elements in each zone, and theoretical models for earth pressure intensity, resultant force, and the point of application of the resultant force were derived. The theoretical results were generally in good agreement with the experimental results and reasonably reflected the distribution characteristics of passive earth pressure in the finite soil mass under inclined support conditions. In particular, the theoretical predictions were in good agreement with the measured values in the principal compression zone in the middle and lower portions of the soil mass. The measured earth pressures at the upper monitoring points were markedly lower because of local wall-soil separation, the inability of cohesionless sand to transmit tensile stress, and wall-soil contact relaxation. Within the experimental validation range, the resultant earth pressure decreased and its point of application generally shifted upward as the support inclination angle increased. In contrast, an increase in the soil friction angle caused the point of application to shift downward. The findings of this study can provide a theoretical reference for the design of inclined support structures in projects adjacent to existing structures.

## 1. Introduction

**Fig 1:**
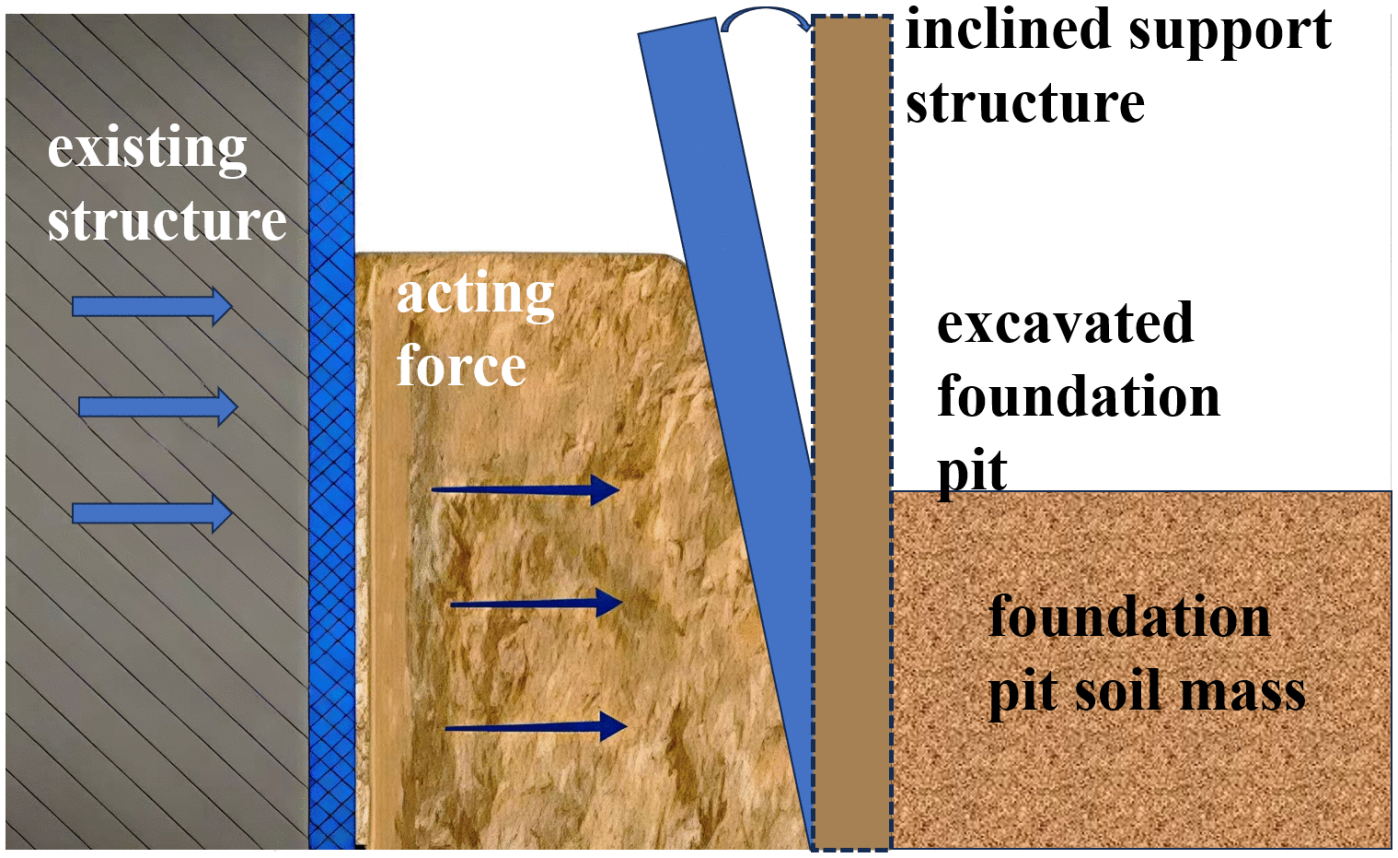
Schematic diagram of inclined support structures adjacent to existing structures.

With the increasing development of urban underground space toward greater depth and higher land-use intensity, foundation pit and tunnel projects adjacent to existing buildings and structures, retaining walls, rock slopes, and other underground structures have become increasingly common. Owing to site constraints and project layout requirements, excavation often needs to be conducted near existing retaining or slope-support structures, resulting in stress redistribution in the soil and changes in the stress state of existing structures. As well as affecting failure surface development and the distribution of lateral earth pressure. Recent studies on narrow backfills and finite soil masses have demonstrated that a limited backfill width can significantly enhance the soil arching effect, causing the earth pressure distribution to deviate markedly from that predicted under the classical semi-infinite backfill assumption [1,2]. For narrow foundation pits, the passive earth pressure acting on support structures is also strongly affected by adjacent boundaries and the confined width of the soil mass [3]. In addition, micro- and meso-scale damage studies of rocks based on computed tomography have further shown that, under complex environmental disturbances, the macroscopic mechanical response of geomaterials is closely related to internal structural damage and its evolution [4,5]. In such projects, inclined support structures, such as raking struts and inclined pile walls, may be adopted, and their mechanical and deformation characteristics differ from those of conventional vertical retaining structures. The intermediate backfill is confined within the limited space between the existing structure and the new support system, rather than behaving as a semi-infinite soil mass as assumed in classical earth pressure theories. Unloading induced by adjacent excavation may cause the existing retaining structure to slide or overturn toward the excavation side. Therefore, the reasonable determination of the earth pressure acting on inclined support structures under such complex conditions has become a key issue in projects adjacent to existing structures. Fig 1 shows a schematic diagram of an inclined support structure adjacent to an existing structure.

At present, earth pressure theories for retaining structures are mainly established within the frameworks of the two classical limit equilibrium theories proposed by Coulomb [6] and Rankine [7]. Rankine’s theory is applicable to the ideal case of a vertical smooth wall back face with a semi-infinite backfill, whereas Coulomb’s theory can account for wall-back inclination and wall roughness. However, Coulomb’s theory assumes a linear distribution of earth pressure and a planar failure surface, and therefore fails to reflect the soil arching effect induced by stress deflection in real soil masses, as well as the resulting nonlinear distribution characteristics of earth pressure. Terzaghi [8] confirmed the existence of the soil arching effect through trapdoor tests, demonstrating that internal stresses in soil can be redistributed due to deformation constraints.

Regarding the soil arching effect, Handy [9] established a minor principal stress trajectory model and proposed a theoretical analysis method based on catenary-shaped soil arches, pointing out that soil arching can lead to a distinctly nonlinear distribution of earth pressure on retaining structures. On this basis, Paik and Salgado [10] further considered wall-soil friction and the variation of soil stress, and proposed an improved method for calculating the active earth pressure coefficient. For narrow backfill conditions, Khosravi et al. [1] developed an active earth pressure model incorporating the soil arching effect, indicating that as the backfill width decreases, soil arching is significantly enhanced and the nonlinear characteristics of lateral earth pressure become more pronounced. Schmüdderich et al. [11] clarified the importance of soil dilatancy and stress paths from the perspectives of the flow rule and limit equilibrium. Almasi [12] further confirmed through numerical analysis of soldier pile retaining wall systems that soil arching in a confined space can cause pronounced nonuniformity in lateral earth pressure.

Regarding finite soil masses, Bashir and Basha [13] investigated the active earth pressure and failure mechanism of narrow-backfill retaining walls under rotational movement through model tests. Li et al. [2] proposed an analytical solution for passive earth pressure considering the finite-width effect, demonstrating that the earth pressure distribution under finite soil mass conditions differs significantly from that predicted by classical Rankine theory. Hu et al. [14] established a displacement-dependent passive earth pressure model for finite soil mass behind cantilever retaining walls, revealing the coupling effect between finite width and wall displacement. Fan et al. [15] proposed an analytical solution for displacement-dependent passive earth pressure and clarified the relationship between displacement functions and pressure distributions. In addition, Shwan [16] investigated the passive earth pressure of unsaturated soil from the perspective of hydraulic-mechanical hysteresis, indicating that the stress path and moisture condition of unsaturated soil significantly affect the development of passive resistance. Shiau et al. [17] further analyzed the influence of spatial variability in soil parameters on passive earth pressure from a probabilistic perspective, showing that the earth pressure problem in finite soil masses involves considerable uncertainty. In terms of experimental studies, many scholars [18–23] have also conducted extensive research on finite soil masses. In addition, studies involving complex geological media have shown that engineering responses are often governed by the coupled effects of material properties, boundary conditions, and external disturbances. Li et al. [24–26] investigated fracturing-fluid leakoff in shale reservoirs, particle sedimentation in CO_2_ fracturing fluids, and wellhead stability during hydrate reservoir development, respectively, highlighting the importance of accounting for complex medium characteristics and boundary effects in mechanical analyses. These studies provide methodological insights for analyzing earth pressure in finite soil masses adjacent to existing structures.

In summary, although significant progress has been made in existing studies on the soil arching effect, finite soil masses, and earth pressure under non-limit states, several limitations remain. Most previous studies have idealized the support structure as a vertical wall back face and have not systematically considered the combined effects of sliding failure of the existing structure, support inclination, the morphology of principal stress trajectories within the finite soil mass, failure surface development, and soil arching on earth pressure. Few studies have incorporated the sliding failure of existing structures, a typical instability mode in adjacent construction, into a unified earth pressure analysis framework.

To address the above limitations, five groups of model tests were conducted in at a fixed width-to-height ratio of B/H = 0.95. The failure process of the soil mass during the model tests was observed using PIV, and the morphology of the failure surfaces was discussed. Considering the practical loading requirements of inclined support structures in adjacent construction projects, a theoretical earth pressure calculation model is proposed for a finite cohesionless soil mass behind the wall, in which the inclination of the support structure, the finite soil mass width, and the soil arching effect are simultaneously considered. Based on the failure surface characteristics observed in the tests, the finite soil mass is divided into several discrete bodies using linear failure surfaces. Equilibrium differential equations are established for horizontal differential elements, from which theoretical models for earth pressure intensity, resultant force, and its point of application are derived. It should be noted that the conclusions of this study are primarily applicable to the experimental conditions with a B/H ratio close to 0.95 and should not be extrapolated excessively to other width-to-height ratios.

## 2. Physical model tests

### 2.1. Physical model test design

The physical model tests were conducted using a custom-built model box comprising a box body, a loading system, an earth pressure measurement system, and an image acquisition system.

**Fig 2:**
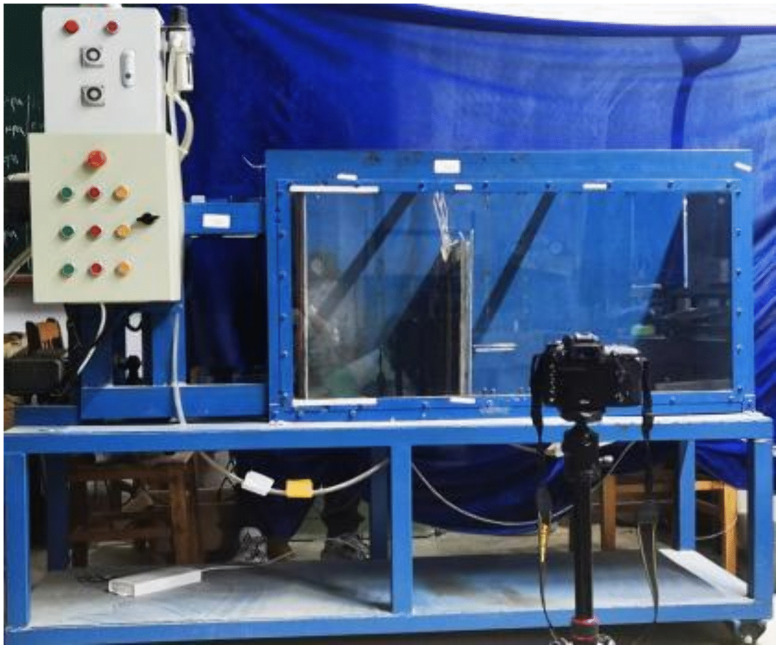
Custom-built model box.

The main body of the model box measured 1200 mm × 400 mm × 700 mm in length, width, and height, respectively. A rigid retaining plate was installed on each side of the box to simulate the adjacent existing structure and the inclined retaining wall. The left retaining plate was 20 mm thick and connected to the loading system; it could be pushed by an adjustable motor to simulate the failure process of the soil mass in the model box. The right retaining plate was 16 mm thick, with five slotted openings arranged along its central vertical line. Earth pressure cells were installed in these openings to measure the variation in earth pressure within the intermediate soil mass during failure. The loading system consisted of two independently controlled motors installed at the upper and lower positions. For image acquisition, a high-speed camera was used to capture images of the soil region during the tests, and the captured images were analyzed using particle image velocimetry (PIV) together with GOM Correlate software. The overall experimental setup and geometric configuration are shown in Fig 2.

The soil sample used in these tests was Dongting Lake sand. After washing, drying, and sieving, sand particles with sizes ranging from 0.075 mm to 0.63 mm were selected as the test soil. The physical and mechanical parameters of the test soil were obtained through direct shear tests and other auxiliary tests, as listed in Table 1.

**Table 1:** Physical and mechanical parameters of the test soil.

Density (g/cm³)	Internal friction angle (°)	Cohesion (kPa)	Wall-soil friction angle (°)	Porosity	Particle size range (mm)
1.45	36	0	22.8	0.65 ~ 0.68	0.075 ~ 0.63

In the tests, the top of the support structure was inclined at five angles, namely 0°, 5°, 10°, 15°, and 20° [27,28]. The backfill height was 400 mm, the base width was 380 mm, and the excavation depth was 200 mm for all test cases, so as to investigate the deformation behavior of the soil mass under different support inclination angles. The model test groups are listed in Table 2.

**Table 2:** Model test groups.

Test No.	Backfill height (mm)	Backfill width (mm)	Width-to-height ratio	Inclination angle (°)
**Test 1**	400	380	0.95	0
**Test 2**	400	380	0.95	5
**Test 3**	400	380	0.95	10
**Test 4**	400	380	0.95	15
**Test 5**	400	380	0.95	20

The test procedure was as follows:

1. Pre-test preparation: The required equipment, such as earth pressure cells and image acquisition devices, was prepared. The right retaining wall was assembled and adjusted to the specified inclination angle. The operating condition of the left-side motor was checked to ensure test safety.
2. Soil placement: The test soil was placed in layers and slightly compacted.
3. Displacement loading: The motor was activated to push the left retaining plate rightward at a constant rate of 0.2 mm/s. The loading was stopped when the preset maximum displacement of 600 mm was reached or when excessive deformation occurred in the right retaining plate.
4. Image acquisition and analysis: The image acquisition system continuously captured images at a rate of one image per second. The captured images were analyzed using the PIV-based GOM Correlate software to observe the failure surfaces, and the earth pressure data were subsequently processed.
5. Repetition of tests for error reduction: After each test, all the soil was removed, and the inclination angle of the right retaining plate or the backfill width was changed for subsequent tests. Each support inclination angle was tested in triplicate, and the earth pressure results were averaged over the repeated tests.

### 2.2. Experimental results and analysis

Test Nos. 1 ~ 5 had a soil width and height of 380 mm and 400 mm, respectively, with inclination angles of 0°, 5°, 10°, 15°, and 20° toward the finite backfill side. The excavation depth was 200 mm in all five test groups. The images captured by the high-speed camera were analyzed using GOM Correlate software based on the PIV technique, together with the earth pressure data measured by the earth pressure cells.

#### 2.2.1. Analysis of failure surface characteristics.

**Fig 3:**
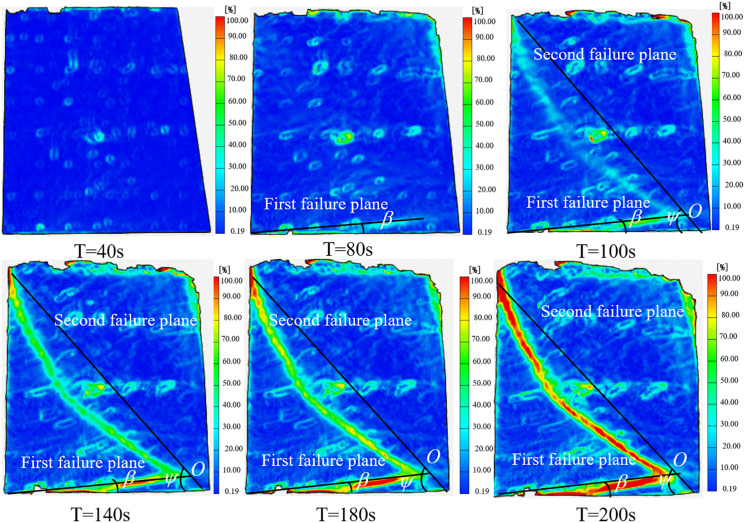
Development of failure surfaces in the soil mass at B/H = 0.95 and an inclination angle of 20°.

Due to space limitations, the case with a support inclination angle of 20° is discussed as an example. As shown in the Fig 3, the stress field within the soil mass was initially in equilibrium. The PIV contours indicate extremely low shear strain increments throughout the soil mass, with no evident displacement gradients or shear bands. As the slope support slid rightward, the finite soil mass between the supports gradually entered a passive compression state. A first failure surface then appeared, exhibiting an approximately straight geometry with a failure angle of about 11°. At T = 100 s, the development of this failure surface ceased. As deformation continued, a second failure surface gradually developed from the top of the slope support to the base of the inclined support structure. This surface exhibited a curved profile with a failure angle of approximately 40°. With further increases in the imposed displacement, the shear bands in the contours became wider, indicating that the internal sliding deformation of the soil mass evolved from a single straight failure surface into a combination of multiple connected line segments. Consequently, the sliding soil wedge developed an asymmetric polygonal profile.

**Fig 4:**
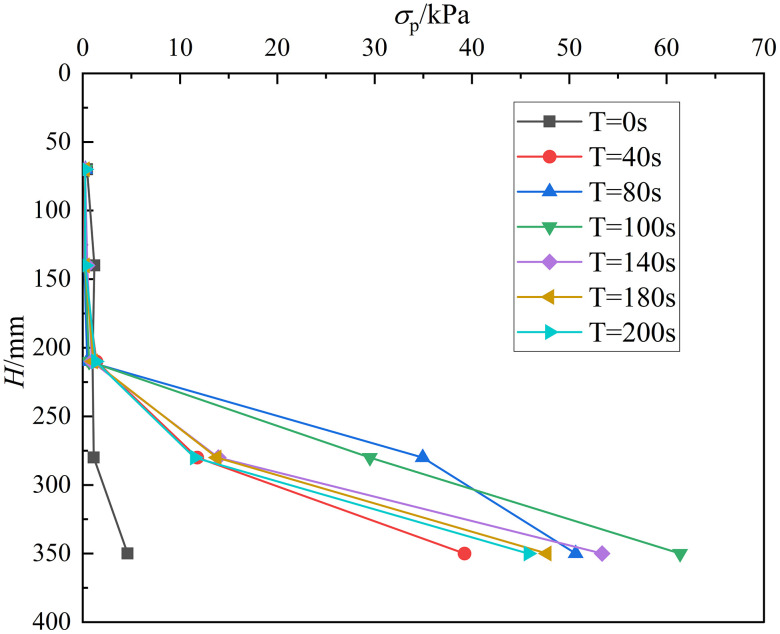
Variation of earth pressure at different times for the soil mass at B/H = 0.95 and an inclination angle of 20°.

As shown in the Fig 4, the earth pressure variations under different inclination angles of the inclined support structure are plotted against the translational displacement time of the existing structure on the left. At T = 0 s, the left steel plate had not yet moved, and the measured values therefore represented the at-rest earth pressure. The earth pressure increased with depth and was generally consistent with the values calculated using the at-rest earth pressure equation. As the deformation of the existing support structure increased, the earth pressure along the wall height gradually increased. The earth pressure reached its maximum value at T = 100 s. The PIV contours show that the second failure surface appeared at this time and became increasingly distinct as the failure process continued. Based on the combined PIV observations and earth pressure measurements, the finite soil mass was considered to have reached the limit state at T = 100 s.

**Fig 5:**
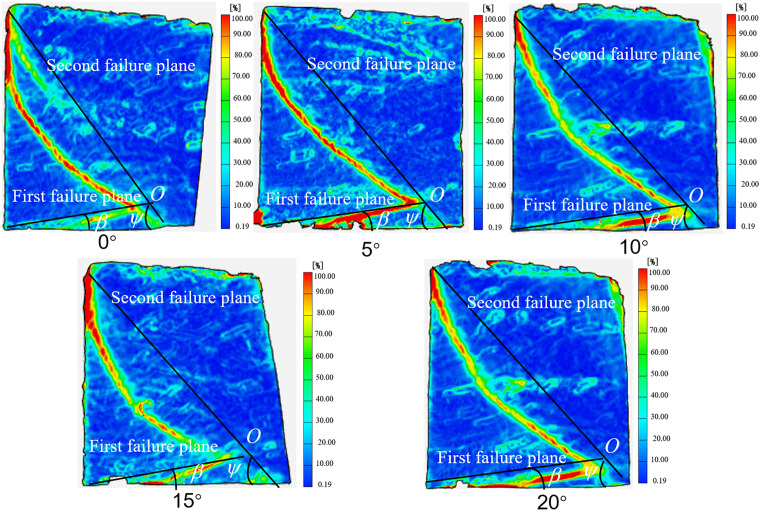
Shear strain contours at the limit state.

A comparative analysis was conducted based on the PIV images and earth pressure measurements obtained from Tests 1 ~ 5. In this study, the limit state was defined as the state at which the primary shear bands became essentially continuous and the resultant earth pressure reached or approached its peak value. The corresponding wall displacement was adopted as a unified quantitative criterion. Based on this criterion, shear strain contours at the limit state under different inclination angles were selected for PIV-based analysis, as shown in Fig 5.

As shown in Fig 5, continuous arc-shaped shear strain bands were formed in the soil mass at the limit state under all inclination conditions, indicating that the failure mode was dominated by progressive shear failure and was characterized by the intersection of two failure surfaces. As the support inclination angle increased from 0° to 20°, the overall orientation of the shear band changed only slightly, whereas differences were observed in its curvature, degree of local strain concentration, and deflection characteristics in the middle and lower regions. This indicates that the inclined support structure mainly affected the details of failure evolution, without changing the fundamental instability mode of the soil mass.

When sliding failure occurred in the left-side support, the first failure surface, approximately linear in shape, initiated from the wall heel of the left support and developed obliquely upward toward the right-side support, where its development was arrested. As failure progressed, a second failure surface began to form from the top of the left existing support structure and extended toward the inclined support structure on the right side until it became fully connected. At the initial loading stage, the shear band first initiated from the wall heel at the left loading end and propagated obliquely upward to the right at a passive failure angle of approximately 8° ~ 20°. Subsequently, owing to the constraint imposed by the rigid boundary on the right side, the failure mode changed, and the second failure surface initiated from the top of the left side and developed toward the lower-right corner. In the case of 0° inclination, two secondary failure surfaces were observed. The second failure surface exhibited an inclination angle of approximately 35° ~ 42°.The second failure surface then extended toward the termination point of the first failure surface, eventually forming an asymmetric polygonal sliding wedge bounded by two intersecting failure surfaces: one extending from the left wall base to the contact point on the right wall body, and the other extending from the left ground surface to the lower part of the right side. This dynamic evolution process indicates that soil failure in a narrow adjacent space is strongly constrained by the boundaries, and the final sliding soil wedge exhibits an asymmetric polygonal shape.

#### 2.2.2. Earth pressure analysis.

For the sand specimen with a width-to-height ratio of B/H = 0.95, model tests were conducted under constant-rate translational pushing of the left-side adjacent existing structure, while the inclination angles of the right retaining wall were set to 0°, 5°, 10°, 15°, and 20°, respectively. Five earth pressure cells, T1 ~ T5, were arranged sequentially along the height of the retaining wall from top to bottom.

**Fig 6:**
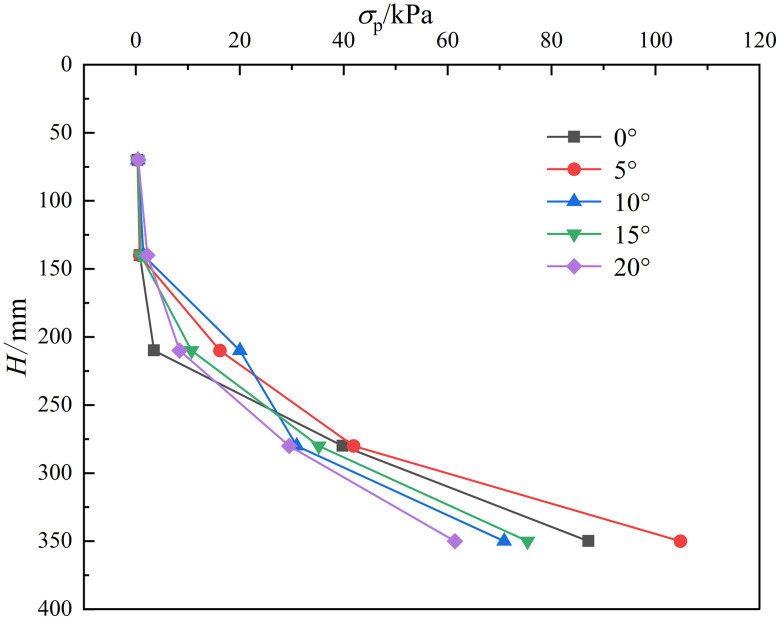
Limit earth pressure values under different inclination angles.

As shown in Fig 6. The earth pressure along the height of the inclined support structure exhibited a markedly nonuniform distribution, with stress highly concentrated in the lower region of the retaining wall. Among the sensors, T5 at the wall base recorded the largest peak earth pressure, the fastest growth rate, and the most pronounced post-peak attenuation, indicating that this region was the primary zone resisting the pushing-induced earth pressure. This observation is fully consistent with the PIV results, which showed that stress concentration first occurred at the slope toe and that shear deformation was most intense in this region. The T4 sensor in the middle-lower part of the retaining wall showed the second strongest earth pressure response, following an evolution pattern similar to that of T5; however, both its peak value and variation amplitude were significantly lower than those of T5. In contrast, the earth pressure values recorded by the T1 ~ T2 sensors in the middle-upper part of the retaining wall were relatively low and remained generally stable throughout the test, without obvious increasing or decreasing trends. This indicates that the additional stress induced by the pushing action was mainly borne by the lower part of the retaining wall.

Because the intermediate backfill was sand and excavation was carried out on the right side, the sand, as a typical granular material, had negligible cohesion during deformation of the inclined support. Unlike cohesive soil, it could not develop tensile resistance. Therefore, when the upper part of the wall tilted outward, local contact relaxation or even slight separation was likely to occur at the wall-soil interface, leading to a substantial reduction in the effective lateral pressure transmitted to the earth pressure cells in the upper part of the wall.

## 3. Derivation of earth pressure for the finite soil mass

### 3.1. Mechanical model and basic assumptions

**Fig 7:**
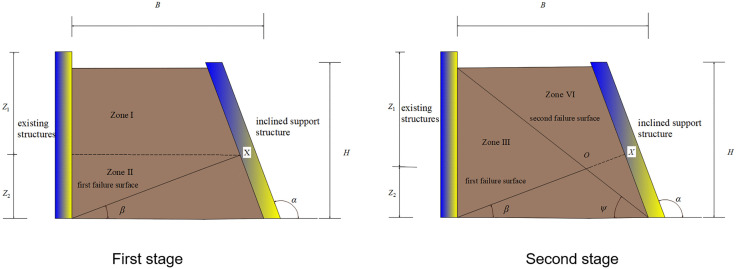
Schematic diagram of the mechanical model.

As shown in Fig 7, a mechanical model was established with the finite soil mass as the object of analysist. The existing structure was located on the left side, the inclined support structure was located on the right side, and the cohesionless soil was confined between them. According to the test results, when sliding failure occurred in the left-side existing structure, the first failure surface initially developed from the bottom of the existing-structure side. As deformation of the left-side support continued, the intermediate finite soil mass underwent further failure, leading to the formation of a second failure surface. Based on the development process of the failure surfaces, the calculation model was divided into two stages. The first stage corresponded to the case in which only the first failure surface appeared, while the second stage corresponded to the case in which both the first and second failure surfaces were formed. When only the first failure surface was present, only the parameters and mechanical properties of the soil itself needed to be considered. When both the first and second failure surfaces were present, the reaction force transferred from the first failure surface during failure needed to be considered in the calculation of the regions divided by the second failure surface.

The retaining wall height is denoted by *H*, the bottom width of the backfill by *B*, the angle between the failure surface behind the wall and the horizontal ground surface by *β*, and the unit weight of the soil by *γ*. The intersection point between the failure surface and the inclined support structure is denoted as point *X*, whose distances from the ground surface and the base are *Z*_1_ and *Z*_2_, respectively. The inclination angle between the right-side inclined support structure and the base is denoted by *α*.

In the first stage, the finite soil mass behind the wall is divided by the first failure surface into two discrete bodies for analysis: Zone I, which is quadrilateral, and Zone II, which is triangular.

In the second stage, the finite soil mass is re-divided according to the configuration of the failure surfaces. The soil mass located to the lower-left side of the second failure surface is defined as Zone III, whereas the soil mass located to the upper-right side of the second failure surface is defined as Zone IV. The intersection point between the first and second failure surfaces is denoted as point *O*.

To simplify the derivation and facilitate the analysis in this study, the following assumptions are made:

1. The first failure surface is assumed to be a straight line passing through the wall heel. [29,30]. Its inclination angle *β*, was determined from the average orientation of the primary shear bands observed in the PIV results.
2. The second failure surface is regarded as a straight line with an inclination angle of *ψ*. Under the experimental condition of B/H = 0.95 considered in this study, *ψ* was determined from the position of the second failure surface observed in the PIV results and the geometric relationship of the finite soil mass.
3. The soil behind the wall is assumed to be homogeneous and cohesionless, with an internal friction angle of *φ*. The external friction angles between the soil and the existing support structure and between the soil and the inclined support structure are denoted by *δ*_1_ and *δ*_2_, respectively.
4. The soil arch formed in the cohesionless soil behind the wall is assumed to be a major principal stress arch, and its shape is taken as a circular arc.

### 3.2. Theoretical derivation

#### 3.2.1. Theoretical derivation for the first stage.

**Fig 8:**
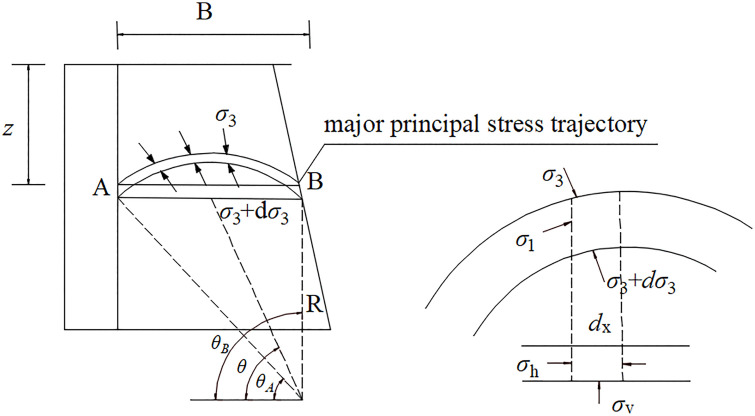
Schematic diagram of major principal stress trajectories in Zone I.

A horizontal differential layer AB is taken at a depth *z* from the soil surface. When AB is located in Zone I, the corresponding condition is shown in Fig 8.

According to the geometric relationship shown in Fig 8, the following relationship can be obtained between the radius *R* of the major principal stress arch in Zone I and the length of AB:


$$\begin{array}{c} R={\frac{B}{cos\theta_{A}+cos\theta }}_{B} \end{array}$$ (1)

**Fig 9:**
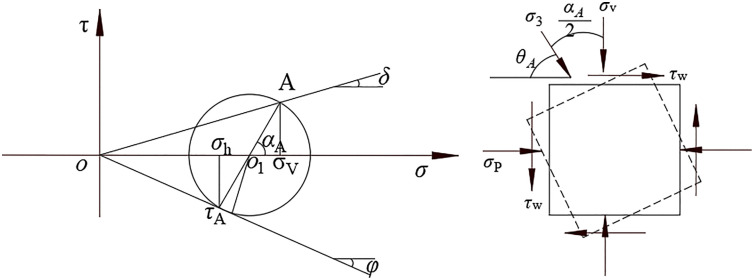
Mohr’s circle of stress at point A.

Mohr’s circle of stress at point A is shown in Fig 9.

According to Mohr’s circle of stress, the principal stress deflection angles on both sides of the finite soil mass can be expressed as:


$$\begin{array}{c} \left\{ \begin{array}{c} @l@\theta_{A}=\frac{\pi }{\text{2}}-\frac{\text{1}}{\text{2}}\left[arcsin\left(\frac{sin\delta_{\text{1}}}{sin\varphi }\right)+\delta_{\text{1}}\right]\\ \theta_{B}=\alpha -\frac{\text{1}}{\text{2}}\delta_{\text{2}}+\frac{\text{1}}{\text{2}}arcsin\left(\frac{sin\delta_{\text{2}}}{sin\varphi }\right) \end{array}\right. \end{array}$$ (2)

Based on the horizontal differential element AB at a depth *z*, as shown in Fig 9, the major principal stress *σ*_3_ is deflected. Let *θ* denote the angle between *σ*_3_ and the horizontal plane at any point along the soil arch. The vertical stress *σ*_v_ and horizontal stress *σ*_h_ at this point can then be expressed as:


$$\begin{array}{c} \left\{ \begin{array}{c} @l@\sigma_{h}=\left({cos}^{\text{2}}\theta +K_{P}{sin}^{\text{2}}\theta \right)\sigma_{\text{3}}\\ \sigma_{v}=\left({sin}^{\text{2}}\theta +K_{P}{cos}^{\text{2}}\theta \right)\sigma_{\text{3}} \end{array}\right. \end{array}$$ (3)

*K*_*P*_: passive earth pressure coefficient.


$$\begin{array}{c} K_{P}=\frac{\sigma_{\text{1}}}{\sigma_{\text{3}}}=\frac{1+sin\varphi }{1-sin\varphi } \end{array}$$ (4)

The average vertical stress ${\overset{―}{\sigma }}_{v}$ on a plane at any depth *z* in Zone I can be expressed as:


$$\begin{array}{c} {\overset{―}{\sigma }}_{v}=\frac{\int_{\theta_{A}}^{\pi -\theta_{B}}dV}{B}=\frac{\int_{\theta_{A}}^{\pi -\theta_{B}}\sigma_{v}Rsin\theta }{B}d\theta =\sigma_{\text{3}}+\frac{{cos}^{\text{3}}\theta_{A}+{cos}^{\text{3}}\theta_{B}}{3\left(cos\theta_{A}+cos\theta_{B}\right)}\left(K_{P}-1\right)\sigma_{\text{3}} \end{array}$$ (5)

Therefore, the passive lateral earth pressure coefficient on the side adjacent to the existing structure, *K*_pwn1_, can be obtained as:


$$\begin{array}{c} K_{pwn1}=\frac{\sigma_{h}}{{\overset{―}{\sigma }}_{v}}=\frac{{cos}^{\text{2}}\theta_{A}+K_{\text{p}}{sin}^{\text{2}}\theta_{A}}{1+\frac{\left({cos}^{\text{3}}\theta_{A}-{cos}^{\text{3}}\theta_{B}\right)}{3\left(cos\theta_{A}-cos\theta_{B}\right)}\left(K_{\text{p}}-1\right)} \end{array}$$ (6)

**Fig 10:**
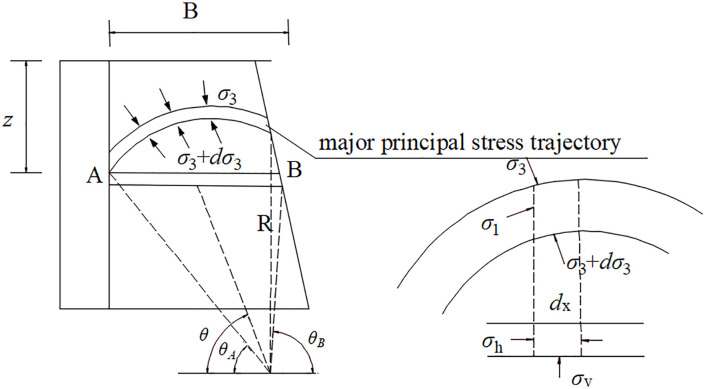
Schematic diagram of major principal stress trajectories in Zone II.

When *z* is located in Zone II, the corresponding horizontal differential layer is denoted by CD, as shown in Fig 10.

According to the geometric relationship shown in Fig 10, the following relationship can be obtained between the radius *R* of the major principal stress arch in Zone II and the distance between points C and D:


$$\begin{array}{c} R=\frac{B}{cos\theta_{C}-cos\theta_{D}} \end{array}$$ (7)

The calculation procedure for Zone II is the same as that for Zone I. The principal stress deflection angles on both sides of the finite soil mass in Zone II are given as:


$$\begin{array}{c} \left\{ \begin{array}{c} @l@\theta_{C}=\frac{\pi }{\text{2}}-\frac{\text{1}}{\text{2}}\left[arcsin\left(\frac{sin\delta_{\text{2}}}{sin\varphi }\right)+\delta_{\text{2}}\right]\\ \theta_{D}=\frac{\pi }{\text{4}}+\frac{\varphi }{\text{2}}+\beta \end{array}\right. \end{array}$$ (8)

The average vertical stress ${\overset{―}{\sigma }}_{v}$ on a plane at any depth *z* in Zone II can be expressed as:


$$\begin{array}{c} {\overset{―}{\sigma }}_{v}=\frac{\int_{\theta_{C}}^{\theta_{D}}dV}{B}=\frac{\int_{\theta_{C}}^{\theta_{D}}\sigma_{v}Rsin\theta }{B}d\theta =\sigma_{\text{3}}\left[1+\frac{{cos}^{\text{3}}\theta_{C}-{cos}^{\text{3}}\theta_{D}}{3\left(cos\theta_{C}-cos\theta_{D}\right)}\left(K_{P}-1\right)\right] \end{array}$$ (9)

The passive lateral earth pressure coefficient on the side adjacent to the existing structure in Zone II, *K*_pwn2_, is given by:


$$\begin{array}{c} K_{pwn2}=\frac{\sigma_{h}}{{\overset{―}{\sigma }}_{v}}=\frac{{cos}^{\text{2}}\theta_{C}+K_{\text{p}}{sin}^{\text{2}}\theta_{C}}{1+\frac{\left({cos}^{\text{3}}\theta_{C}-{cos}^{\text{3}}\theta_{D}\right)}{3\left(cos\theta_{C}-cos\theta_{D}\right)}\left(K_{\text{p}}-1\right)} \end{array}$$ (10)

**Fig 11:**
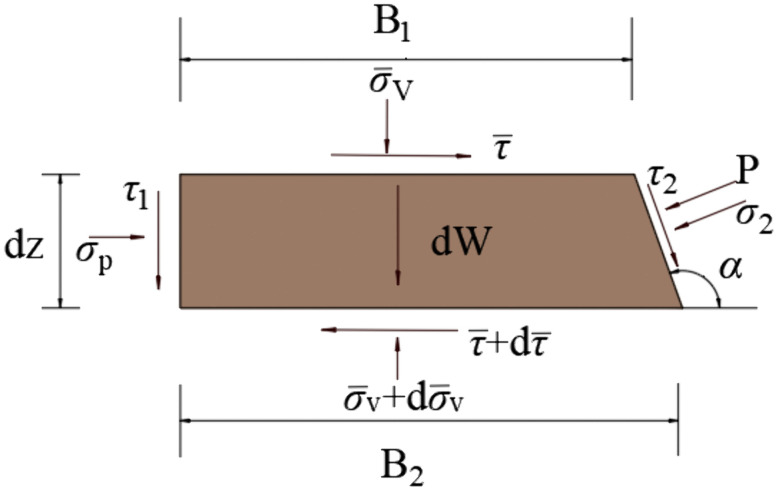
Mechanical model of a differential element in Zone I under passive failure. In the figure: *d*W: self-weight of the soil differential element; *σ*_p_: horizontal stress on the side of the existing structure; *σ*_2_: horizontal stress on the side of the inclined support structure; *σ*_v_: average reaction force transmitted from the upper differential element to the lower differential element; *τ*_1_: shear stress on the side of the existing structure; *τ*_2_: shear stress on the side of the inclined support structure; $\overset{―}{\tau }$: average shear stress transmitted from the upper differential element to the lower differential element; *dz*: height of the differential element; *P*: pressure exerted by the inclined support structure on the differential element.

The quadrilateral region above the intersection point *X* of the first failure surface, namely Zone I, is selected as the object of analysis. A horizontal differential element at an arbitrary depth *z* above point *X* is taken for force analysis, where z < Z_1_. In this case, the differential element is located in Zone I, and its mechanical model is shown in Fig 11.

The widths of the upper and lower bases of the differential element are given by:


$$\begin{array}{c} B_{\text{1}}=B+\left(H-z\right)cot\alpha \end{array}$$ (11)

$$\begin{array}{c} B_{\text{2}}=B_{\text{1}}-dzcot\alpha \end{array}$$ (12)

During the deformation process, the weight of the soil in this region is assumed to remain unchanged and can be expressed as:


$$\begin{array}{c} dW=\frac{\gamma }{\text{2}}\left(B_{\text{1}}+B_{\text{2}}\right)dz \end{array}$$ (13)

Where *γ* is the unit weight of the soil.

Combining Equations (11) and (12), and neglecting higher-order terms, it can be simplified as:


$$\begin{array}{c} dW=\gamma B_{\text{1}}dz \end{array}$$ (14)

Based on the mechanical model, differential equilibrium equations are established for the differential element in the horizontal and vertical directions.

In the horizontal direction, by setting Σ*X* = 0:


$$\begin{array}{c} \begin{array}{c} \sigma_{p}dz+B_{\text{1}}\overset{―}{\tau }+\tau_{\text{2}}cos\left(\pi -\alpha \right)dz-B_{\text{2}}\left(\overset{―}{\tau }+d\overset{―}{\tau }\right)-\\ \sigma_{\text{2}}sin\left(\pi -\alpha \right)dz-Psin\left(\pi -\alpha \right)dz=0 \end{array} \end{array}$$ (15)

In the vertical direction, by setting Σ*Y* = 0:


$$\begin{array}{c} \begin{array}{c} B_{\text{1}}{\overset{―}{\sigma }}_{v}+dW+\tau_{\text{2}}sin\left(\pi -\alpha \right)dz+\sigma_{\text{2}}cos\left(\pi -\alpha \right)dz\\ +Pcos\left(\pi -\alpha \right)dz-B_{\text{2}}\left({\overset{―}{\sigma }}_{v}+d{\overset{―}{\sigma }}_{v}\right)+\tau_{\text{1}}dz=0 \end{array} \end{array}$$ (16)

The relationship between the shear stress and the horizontal stress on the side of the existing structure is given by:


$$\begin{array}{c} \tau_{\text{1}}=\sigma_{p}tan\delta_{\text{1}} \end{array}$$ (17)

The relationship between the shear stress and the horizontal stress on the side of the inclined support structure is given by:


$$\begin{array}{c} \tau_{\text{2}}=\sigma_{\text{2}}tan\delta_{\text{2}} \end{array}$$ (18)

According to the soil arching effect, the following relationship can be obtained:


$$\begin{array}{c} \sigma_{p}=K_{pwn1}{\overset{―}{\sigma }}_{v} \end{array}$$ (19)

Because the shear stress is relatively small during translational movement of the support structure, it can be neglected in the calculation. By combining Equations (14)–(19) and neglecting higher-order terms, the following expression can be obtained:


$$\begin{array}{c} \frac{d{\overset{―}{\sigma }}_{v}}{dz}=\gamma +\frac{{\overset{―}{\sigma }}_{v}A_{\text{1}}-PA_{\text{2}}}{B_{\text{1}}} \end{array}$$ (20)

In the formula:


$$\begin{array}{c} \begin{array}{c} A_{\text{1}}=K_{pwn1}\left[tan\delta_{\text{1}}-tan\left(\alpha +\delta_{\text{2}}\right)\right]+cot\alpha \\ A_{\text{2}}=cos\alpha -sin\alpha tan\left(\alpha +\delta_{\text{2}}\right) \end{array} \end{array}$$

**Fig 12:**
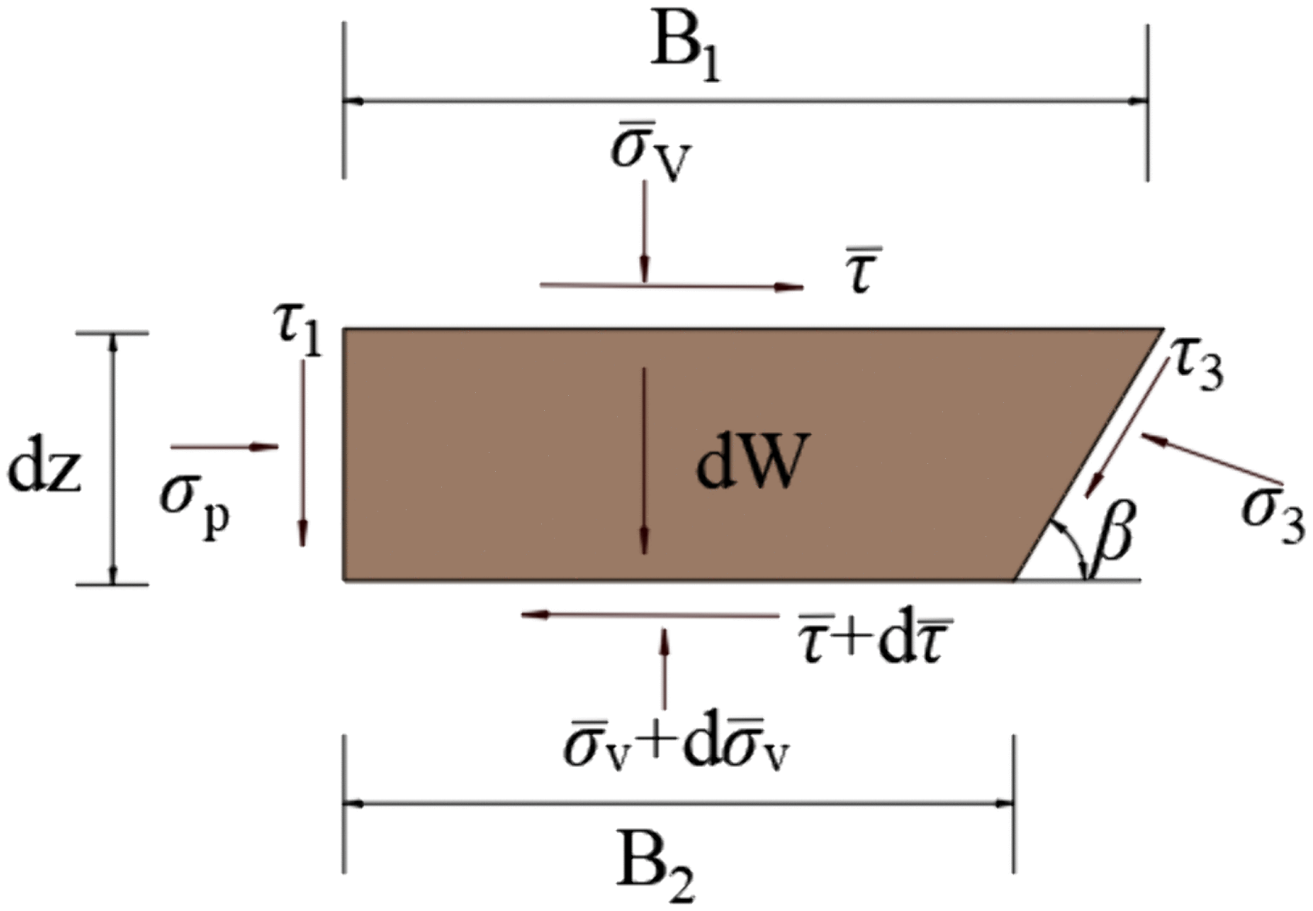
Mechanical model of a differential element in Zone II under passive failure. In the figure: *τ*_3_: shear stress exerted by the first failure surface on the soil differential element; *σ*_3_: reaction force exerted by the first failure surface on the soil differential element.

A horizontal differential element at an arbitrary depth *z* below point *X* is taken for force analysis, where *z* > *Z*_1_. In this case, the differential element is located in Zone II, and its mechanical model is shown in Fig 12.

The relationship between the shear stress exerted by the first failure surface on the soil differential element and the corresponding reaction force is given by:


$$\begin{array}{c} \tau_{\text{3}}=\sigma_{\text{3}}tan\varphi_{p} \end{array}$$ (21)

Where *φ*_p_ is the internal friction angle of the soil under the passive state.

The widths of the upper and lower bases of the differential element in Zone II, as well as the weight of the differential element, are given by:


$$\begin{array}{c} B_{\text{1}}=\left(H-z\right)cot\beta \end{array}$$ (22)

$$\begin{array}{c} B_{\text{2}}=B_{\text{1}}-dzcot\beta \end{array}$$ (23)

$$\begin{array}{c} dW=\frac{\gamma }{\text{2}}\left(B_{\text{1}}+B_{\text{2}}\right)dz \end{array}$$ (24)

Based on the mechanical model, differential equilibrium equations are established for the differential element in the horizontal and vertical directions.

In the horizontal direction, by setting Σ*X* = 0:


$$\begin{array}{c} \sigma_{p}dz+B_{\text{1}}\overset{―}{\tau }-B_{\text{2}}\left(\overset{―}{\tau }+d\overset{―}{\tau }\right)-\sigma_{\text{3}}sin\beta dz-\tau_{\text{3}}cos\beta dz=0 \end{array}$$ (25)

In the vertical direction, by setting Σ*Y* = 0:


$$\begin{array}{c} B_{\text{1}}{\overset{―}{\sigma }}_{v}+\tau_{\text{1}}dz-B_{\text{2}}\left({\overset{―}{\sigma }}_{v}+d{\overset{―}{\sigma }}_{v}\right)+dW+\tau_{\text{3}}sin\beta dz-\sigma_{\text{3}}cos\beta dz=0 \end{array}$$ (26)

Combining the above equations and neglecting higher-order terms, the expression can be simplified as:


$$\begin{array}{c} \frac{d{\overset{―}{\sigma }}_{v}}{dz}=\gamma +\frac{{\overset{―}{\sigma }}_{v}A_{\text{3}}}{B_{\text{1}}} \end{array}$$ (27)

In the formula:


$$\begin{array}{c} A_{\text{3}}=K_{pwn2}tan\delta_{\text{1}}+cot\beta -K_{pwn2}cot\left(\varphi_{p}+\beta \right) \end{array}$$

#### 3.2.2. Theoretical derivation for the second stage.

**Fig 13:**
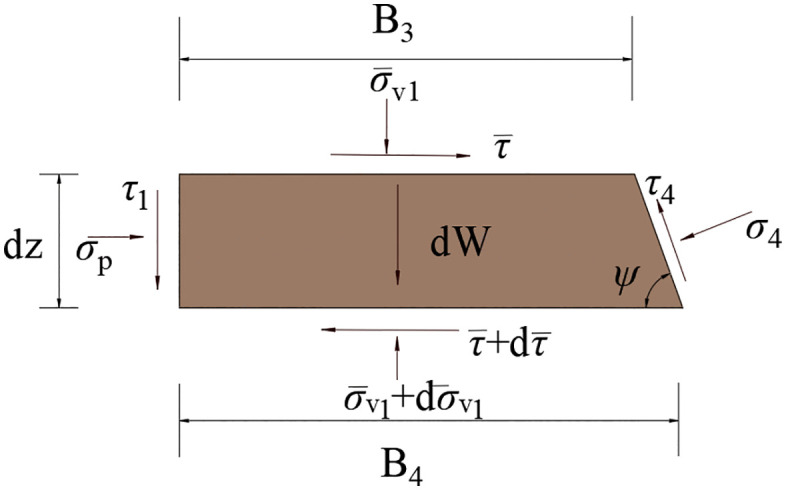
Mechanical model of Zone III above point O. In the figure: *σ*_4_: reaction force exerted by the second failure surface on the soil differential element; *τ*_4_: shear stress exerted by the second failure surface on the soil differential element; ${\overset{―}{\sigma }}_{v1}$: average reaction force transmitted from the upper differential element to the lower differential element.

A horizontal differential element in Zone III above point *O*, located at a depth *z* from the ground surface, is selected for analysis. Its mechanical model is shown in Fig 13.

The relationship between the shear stress and the horizontal stress on the side of the existing structure is given by:


$$\begin{array}{c} \tau_{\text{1}}=\sigma_{p}tan\delta_{\text{1}} \end{array}$$ (28)

The relationship between the shear stress exerted by the second failure surface on the soil differential element and the corresponding reaction force is given by:


$$\begin{array}{c} \tau_{\text{4}}=\sigma_{\text{4}}tan\varphi_{p} \end{array}$$ (29)

The widths of the upper and lower bases of the differential element in Zone III above point *O*, as well as the weight of the differential element, are given by:


$$\begin{array}{c} B_{\text{3}}=zcot\psi \end{array}$$ (30)

$$\begin{array}{c} B_{\text{4}}=B_{\text{3}}+dzcot\psi \end{array}$$ (31)

$$\begin{array}{c} dW=\frac{\gamma }{\text{2}}\left(B_{\text{3}}+B_{\text{4}}\right)dz \end{array}$$ (32)

Based on the mechanical model, differential equilibrium equations are established for the differential element in the horizontal and vertical directions.

In the horizontal direction, by setting Σ*X* = 0:


$$\begin{array}{c} \sigma_{p}dz+B_{\text{3}}\overset{―}{\tau }-B_{\text{4}}\left(\overset{―}{\tau }+d\overset{―}{\tau }\right)-\sigma_{\text{4}}sin\psi dz-\tau_{\text{4}}cos\psi dz=0 \end{array}$$ (33)

In the vertical direction, by setting Σ*Y* = 0:


$$\begin{array}{c} B_{\text{3}}{\overset{―}{\sigma }}_{v1}+\tau_{\text{1}}dz-B_{\text{4}}\left({\overset{―}{\sigma }}_{v1}+d{\overset{―}{\sigma }}_{v1}\right)+dW-\tau_{\text{4}}sin\psi dz+\sigma_{\text{4}}cos\psi dz=0 \end{array}$$ (34)

Following the same calculation procedure described above, the shear stress on the side of the existing support structure is relatively small during translational movement; therefore, the interlayer shear stress can be neglected. By neglecting higher-order terms, the expression can be simplified as:


$$\begin{array}{c} \sigma_{\text{4}}=\frac{\sigma_{p}\left(1-tan\delta_{\text{1}}tan\varphi_{p}\right)-B_{\text{3}}\gamma tan\varphi_{p}}{sin\psi \left(1-{tan}^{\text{2}}\varphi_{p}\right)+2tan\varphi_{p}cos\psi } \end{array}$$ (35)

**Fig 14:**
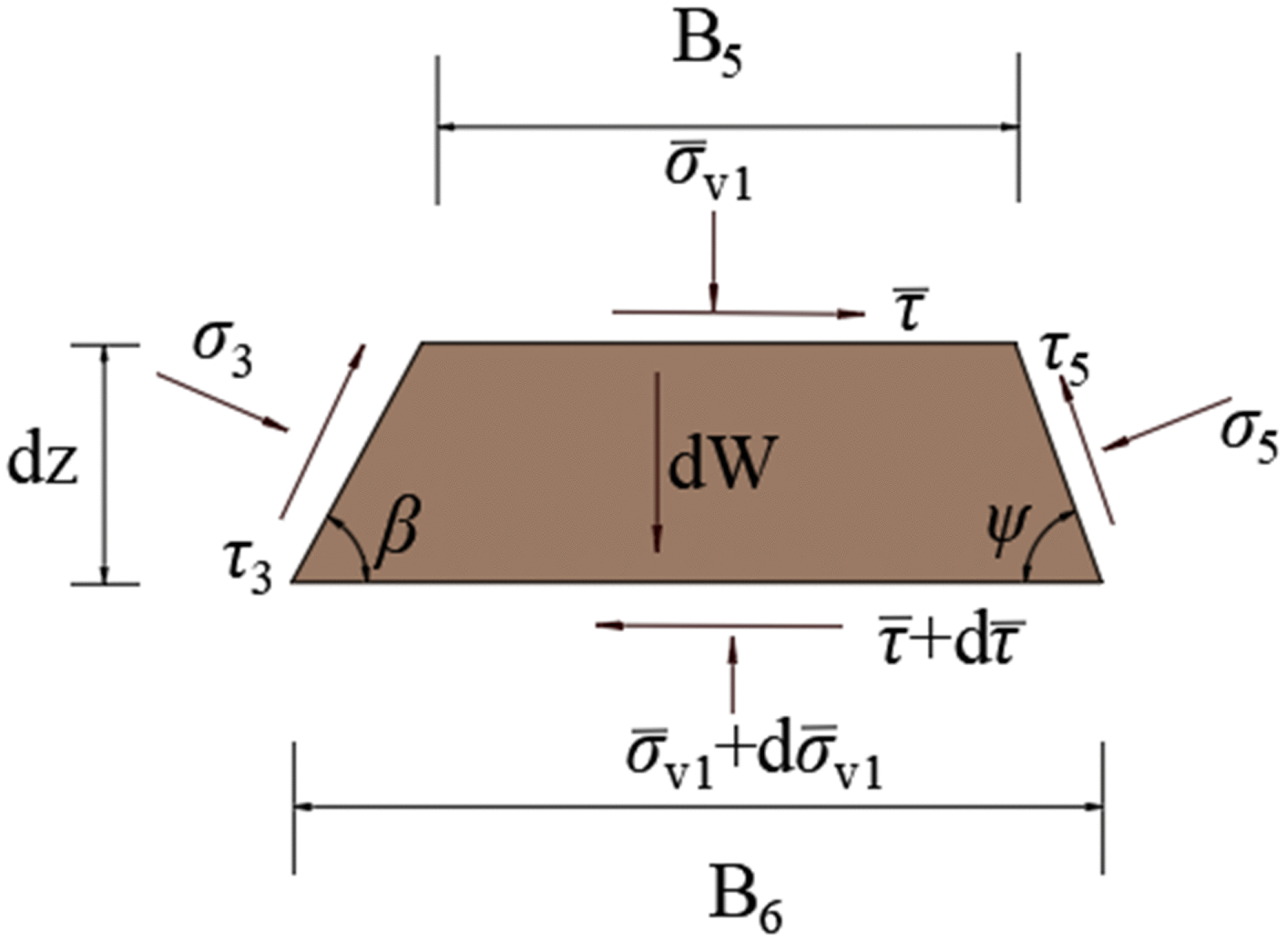
Mechanical model of Zone III below point O and the first failure surface. In the figure: *σ*_3_: reaction force exerted by the first failure surface on the soil differential element; *σ*_5_: reaction force exerted by the second failure surface on the soil differential element; *τ*_3_: shear stress exerted by the first failure surface on the soil differential element; *τ*_5_: shear stress exerted by the second failure surface on the soil differential element.

A horizontal differential element in Zone III below point *O* and the first failure surface, located at a depth *z* from the ground surface, is selected for analysis. Its mechanical model is shown in Fig 14.

The relationship between the shear stress exerted by the first failure surface on the soil differential element and the corresponding reaction force is given by:


$$\begin{array}{c} \tau_{\text{3}}=\sigma_{\text{3}}tan\varphi_{p} \end{array}$$ (36)

The relationship between the shear stress exerted by the second failure surface on the soil differential element and the corresponding reaction force is given by:


$$\begin{array}{c} \tau_{\text{5}}=\sigma_{\text{5}}tan\varphi_{p} \end{array}$$ (37)

The widths of the upper and lower bases of the differential element in Zone III below point *O* and the first failure surface, as well as the weight of the differential element, are given by:


$$\begin{array}{c} \left\{ \begin{array}{c} @l@B_{\text{5}}=B-\left(H-z\right)\left(cot\beta +cot\psi \right)\\ B_{\text{6}}=B_{\text{5}}+\left(cot\beta +cot\psi \right)dz \end{array}\right. \end{array}$$ (38)

$$\begin{array}{c} dW=\frac{\gamma }{\text{2}}\left(B_{\text{5}}+B_{\text{6}}\right)dz \end{array}$$ (39)

Based on the mechanical model, differential equilibrium equations are established for the differential element in the horizontal and vertical directions.

In the horizontal direction, by setting Σ*X* = 0:


$$\begin{array}{c} \sigma_{\text{3}}sin\beta dz+B_{\text{5}}\overset{―}{\tau }-B_{\text{6}}\left(\overset{―}{\tau }+d\overset{―}{\tau }\right)+\tau_{\text{3}}cos\beta dz-\sigma_{\text{5}}sin\psi dz-\tau_{\text{5}}cos\psi dz=0 \end{array}$$ (40)

In the vertical direction, by setting Σ*Y* = 0:


$$\begin{array}{c} \begin{array}{c} B_{\text{5}}{\overset{―}{\sigma }}_{\text{v1}}-\sigma_{\text{3}}cos\beta dz+\tau_{\text{3}}sin\beta dz+dW-\\ B_{\text{6}}\left({\overset{―}{\sigma }}_{\text{v1}}+d{\overset{―}{\sigma }}_{\text{v1}}\right)+\sigma_{\text{5}}cos\psi dz-\tau_{\text{5}}sin\psi dz=0 \end{array} \end{array}$$ (41)

After simplification, the following expression can be obtained:


$$\begin{array}{c} \sigma_{\text{5}}=\frac{\sigma_{\text{3}}A_{\text{4}}-tan\varphi_{p}\gamma B_{\text{5}}}{sin\psi +tan\varphi_{p}\left(2cos\psi -sin\psi tan\varphi_{p}\right)} \end{array}$$ (42)

In the formula:


$$\begin{array}{c} A_{\text{4}}=\left(sin\beta -cos\beta tan\varphi_{p}\right)-tan\varphi_{p}\left(sin\beta tan\varphi_{p}-cos\beta \right) \end{array}$$

**Fig 15:**
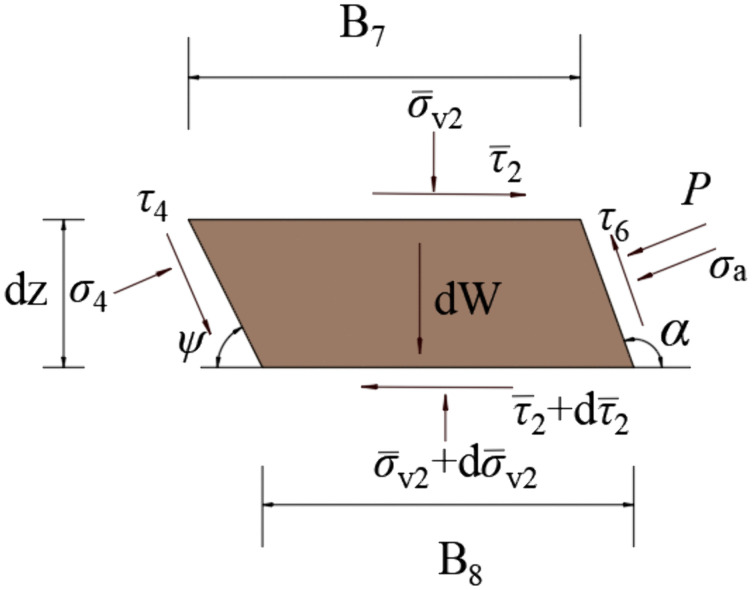
Mechanical model of Zone IV above point O. In the figure: *σ*_*a*_: horizontal stress on the side of the inclined support structure; *σ*_4_: reaction force exerted by the second failure surface on the soil differential element; ${\overset{―}{\sigma }}_{v2}$: average reaction force transmitted from the upper differential element to the lower differential element; ${\overset{―}{\tau }}_{2}$: average shear stress transmitted from the upper differential element to the lower differential element; *τ*_4_: shear stress exerted by the second failure surface on the soil differential element; *τ*_6_: shear stress on the side of the inclined support structure.

A horizontal differential element in Zone IV above point *O*, located at a depth *z* from the ground surface, is selected for analysis. Its mechanical model is shown in Fig 15.

The relationship between the average shear stress and the average reaction force transmitted from the upper differential element to the lower differential element is given by:


$$\begin{array}{c} {\overset{―}{\tau }}_{\text{2}}={\overset{―}{\sigma }}_{v2}tan\varphi_{a} \end{array}$$ (43)

Where *φ*_*a*_ is the internal friction angle of the soil under the active state.

The relationship between the reaction force exerted by the second failure surface on the soil differential element and the shear stress exerted by the second failure surface is given by:


$$\begin{array}{c} \tau_{\text{4}}=\sigma_{\text{4}}tan\varphi_{a} \end{array}$$ (44)

The relationship between the horizontal stress and the shear stress on the side of the inclined support structure is given by:


$$\begin{array}{c} \tau_{\text{6}}=\sigma_{a}tan\delta_{\text{2}} \end{array}$$ (45)

The widths of the upper and lower bases of the differential element, as well as the weight of the differential element, are given by:


$$\begin{array}{c} B_{\text{7}}=B-zcot\psi -\left(H-z\right)cot\left(\pi -\alpha \right) \end{array}$$ (46)

$$\begin{array}{c} B_{\text{8}}=B_{\text{7}}-dzcot\psi +dzcot\left(\pi -\alpha \right) \end{array}$$ (47)

$$\begin{array}{c} dW=\frac{\gamma }{\text{2}}\left(B_{\text{7}}+B_{\text{8}}\right)dz \end{array}$$ (48)

Based on the mechanical model, differential equilibrium equations are established for the differential element in the horizontal and vertical directions.

In the horizontal direction, by setting Σ*X* = 0:


$$\begin{array}{c} \begin{array}{c} \sigma_{a}sin\left(\pi -\alpha \right)dz+Psin\left(\pi -\alpha \right)dz+\tau_{\text{6}}cos\left(\pi -\alpha \right)dz-B_{\text{7}}{\overset{―}{\tau }}_{\text{2}}\\ +B_{\text{8}}\left({\overset{―}{\tau }}_{\text{2}}+d{\overset{―}{\tau }}_{\text{2}}\right)-\sigma_{\text{4}}sin\psi dz-\tau_{\text{4}}cos\psi dz=0 \end{array} \end{array}$$ (49)

In the vertical direction, by setting Σ*Y* = 0:


$$\begin{array}{c} \begin{array}{c} B_{\text{7}}{\overset{―}{\sigma }}_{v2}-B_{\text{8}}\left({\overset{―}{\sigma }}_{v2}+d{\overset{―}{\sigma }}_{v2}\right)+dW+\tau_{\text{4}}sin\psi dz-\sigma_{\text{4}}cos\psi dz\\ +\sigma_{a}cos\left(\pi -\alpha \right)dz+Pcos\left(\pi -\alpha \right)dz-\tau_{\text{6}}sin\left(\pi -\alpha \right)dz=0 \end{array} \end{array}$$ (50)

By combining Equations (43)–(49) and eliminating higher-order terms, the following expression can be obtained:


$$\begin{array}{c} \frac{d{\overset{―}{\sigma }}_{v2}}{dz}=\frac{\gamma B_{\text{7}}-A_{\text{5}}{\overset{―}{\sigma }}_{v2}+\tau_{\text{4}}sin\psi -\sigma_{\text{4}}cos\psi -\sigma_{a}A_{\text{6}}-Pcos\alpha }{B_{\text{7}}} \end{array}$$ (51)

In the formula:


$$\begin{array}{c} \begin{array}{c} A_{\text{5}}=tan\alpha -cot\psi \\ A_{\text{6}}=cos\alpha +tan\delta_{\text{2}}sin\alpha \end{array} \end{array}$$

By combining Equations (49)–(51), the expression can be simplified as:


$$\begin{array}{c} \sigma_{a}=\frac{\sigma_{\text{4}}A_{\text{7}}-Psin\alpha -tan\varphi_{a}\left(\gamma X_{\text{7}}-Pcos\alpha \right)}{sin\alpha \left(1-tan\varphi_{a}tan\delta_{\text{2}}\right)-2tan\delta_{\text{2}}cos\alpha } \end{array}$$ (52)

In the formula:


$$\begin{array}{c} A_{\text{7}}=sin\psi +cos\psi tan\varphi_{a}+tan\varphi_{a}\left(cos\psi -sin\psi tan\varphi_{a}\right) \end{array}$$

**Fig 16:**
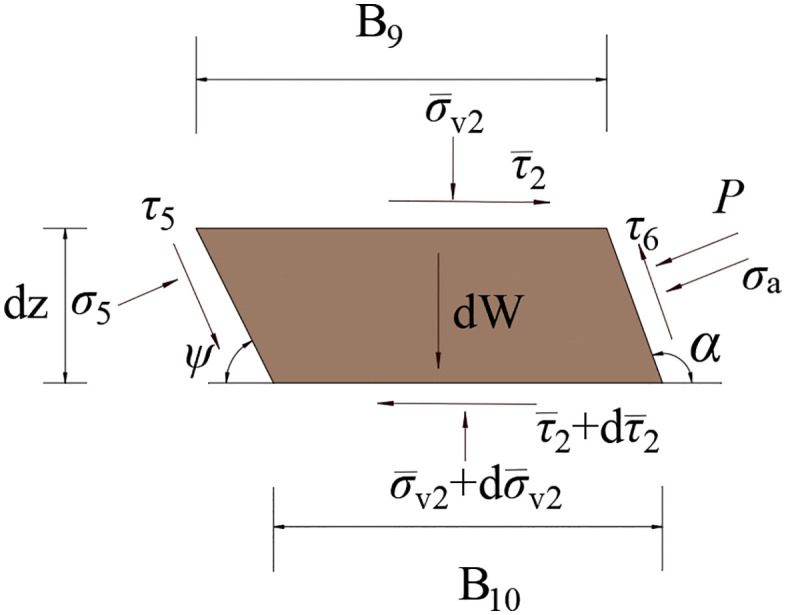
Mechanical model of Zone IV below pointO. In the figure: *σ*_*a*_: horizontal stress on the side of the inclined support structure; *σ*_5_: reaction force exerted by the second failure surface on the soil differential element; *τ*_6_: shear stress on the side of the inclined support structure; *τ*_5_: shear stress exerted by the second failure surface on the soil differential element.

A horizontal differential element in Zone IV below point *O*, located at a depth *z* from the ground surface, is selected for analysis. Its mechanical model is shown in Fig 16.

The relationship between the shear stress exerted by the second failure surface on the soil differential element and the corresponding reaction force is given by:


$$\begin{array}{c} \tau_{\text{5}}=\sigma_{\text{5}}tan\varphi_{a} \end{array}$$ (53)

The widths of the upper and lower bases of the differential element, as well as the weight of the differential element, are given by:


$$\begin{array}{c} B_{\text{9}}=B-zcot\psi -\left(H-z\right)cot\left(\pi -\alpha \right) \end{array}$$ (54)

$$\begin{array}{c} B_{10}=B_{\text{9}}-dzcot\psi +dzcot\left(\pi -\alpha \right) \end{array}$$ (55)

$$\begin{array}{c} dW=\frac{\gamma }{\text{2}}\left(B_{\text{9}}+B_{10}\right)dz \end{array}$$ (56)

Based on the mechanical model, differential equilibrium equations are established for the differential element in the horizontal and vertical directions.

In the horizontal direction, by setting Σ*X* = 0:


$$\begin{array}{c} \begin{array}{c} \sigma_{a}sin\left(\pi -\alpha \right)dz+Psin\left(\pi -\alpha \right)dz+\tau_{\text{6}}cos\left(\pi -\alpha \right)dz-B_{\text{9}}{\overset{―}{\tau }}_{\text{2}}\\ +B_{10}\left({\overset{―}{\tau }}_{\text{2}}+d{\overset{―}{\tau }}_{\text{2}}\right)-\sigma_{\text{5}}sin\psi dz-\tau_{\text{5}}cos\psi dz=0 \end{array} \end{array}$$ (57)

In the vertical direction, by setting Σ*Y* = 0:


$$\begin{array}{c} \begin{array}{c} B_{\text{9}}{\overset{―}{\sigma }}_{v2}-B_{10}\left({\overset{―}{\sigma }}_{v2}+d{\overset{―}{\sigma }}_{v2}\right)+dW+\tau_{\text{5}}sin\psi dz-\sigma_{\text{5}}cos\psi dz\\ +\sigma_{a}cos\left(\pi -\alpha \right)dz+Pcos\left(\pi -\alpha \right)dz-\tau_{\text{6}}sin\left(\pi -\alpha \right)dz=0 \end{array} \end{array}$$ (58)

After simplification, the following expression can be obtained:


$$\begin{array}{c} \sigma_{a}=\frac{\sigma_{\text{5}}A_{\text{7}}-Psin\alpha -tan\varphi_{a}\left(\gamma X_{\text{9}}-Pcos\alpha \right)}{sin\alpha \left(1-tan\varphi_{a}tan\delta_{\text{2}}\right)-2tan\delta_{\text{2}}cos\alpha } \end{array}$$ (59)

In the formula:


$$\begin{array}{c} A_{\text{7}}=sin\psi +cos\psi tan\varphi_{a}+tan\varphi_{a}\left(cos\psi -sin\psi tan\varphi_{a}\right) \end{array}$$

The earth pressure in each soil layer was calculated using the finite difference method. The soil mass was divided into *n* equal layers, each with a thickness ofΔ*z*, and the resultant earth pressure was obtained by summing the earth pressure contributions from layers. The solution was implemented using MATLAB. The calculation was performed as follows:


$$\begin{array}{c} E=\sum_{i=1}^{n}\frac{{\overset{―}{\sigma_{a}}}^{i}}{cos\delta_{\text{2}}}\Delta z \end{array}$$ (60)

In the formula:

E: magnitude of the resultant earth pressure;

*Δz*: height of each differential element.


$$\begin{array}{c} \left\{ \begin{array}{c} @l@M=\sum_{i=1}^{n}{\overset{―}{\sigma_{a}}}^{i}\left(H-z\right)\Delta z\\ h=\frac{M}{E} \end{array}\right. \end{array}$$ (61)

In the formula:

*M*: overturning moment induced by the earth pressure behind the retaining wall;

*z*: depth of the differential element from the backfill surface;

*h*: height of the point of application of the resultant earth pressure above the backfill base.

### 3.3. Comparison of calculated and measured earth pressures

#### 3.3.1. Comparison between theoretical predictions and measured results.

The theoretical predictions obtained using the proposed method were compared with the experimental measurements. The finite soil mass had a width of 380 mm and a height of 400 mm. Five support inclination angles, namely 0°, 5°, 10°, 15°, and 20°, were considered, and the excavation depth was 200 mm. The remaining parameters required for the theoretical calculation were the same as those measured for the test sand, as listed in Table 3.

**Table 3:** Physical and mechanical parameters of the test soil.

width-to-height ratio	Density (g/cm^3^)	Internal friction angle (°)	Cohesion (kPa)	Wall-soil friction angle (°)
0.95	1.45	36	0	22.8

**Fig 17:**
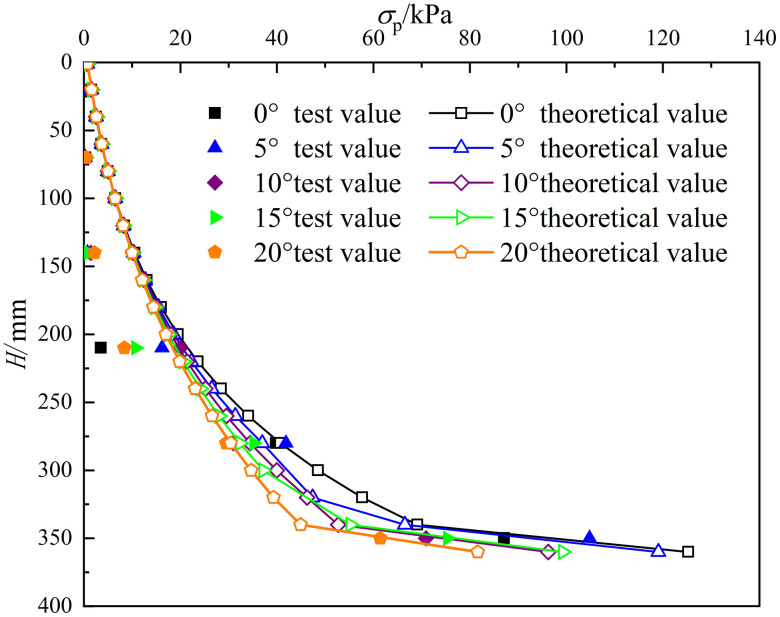
Comparison between measured and theoretical earth pressures under different inclination angles.

No surcharge was applied on the backfill surface. The comparison between the measured limit earth pressure values and the theoretical predictions under different inclination angles is shown in Fig 17.

As shown in Fig 17, the theoretical predictions were compared with the measured values using relative deviations.


$$\begin{array}{c} \eta =\frac{p_{th}-p_{exp}}{p_{exp}}\times 100\% \end{array}$$ (62)

where *p*_th_ and *p*_exp_ denote the theoretical and measured values, respectively; *η* > 0 indicates that the theoretical value is overestimated, whereas *η* < 0 indicates that it is underestimated.

The results indicate that the proposed theoretical model provides good predictive capability at the middle and lower monitoring points, T4 and T5, on the retaining wall. For T4, the relative deviation, *η*, ranged from −11.72% to 11.20% across the five support inclination angles, with a mean absolute relative deviation of 7.22%. For T5, *η* ranged from −11.45% to 11.58%, with a mean absolute relative deviation of 6.72%. Considering the combined set of 10 data points from T4 and T5, the mean absolute relative deviation was 6.97%, indicating that the proposed model can reasonably capture the variation in earth pressure within the primary compression zone in the middle and lower portions of the finite soil mass. The comparison between the experimental results and the theoretical predictions showed good agreement at the T4 and T5 earth pressure cells, whereas relatively larger discrepancies were observed at the remaining three earth pressure cells. Both the theoretical and experimental results exhibited nonlinear distributions, and their overall trends were consistent. The maximum earth pressure occurred at the lowest measuring point. A pronounced variation in the theoretical results appeared in the lowest layer, approximately near the intersection of the first and second failure surfaces.

In contrast, the theoretical predictions at monitoring point T3 exhibited relatively larger deviations. Across the five test conditions, the relative deviation, *η*, ranged from −1.60% to 515.23%, and the theoretical values exceeded the measured values for all cases except the 10° inclination angle. In particular, under the 0°, 15°, and 20° inclination angles, the theoretical values were 515.23%, 78.02%, and 103.99% higher than the measured values, respectively. In this test, he upper three earth pressure cells recorded significantly lower readings, which resulted from the combined effects of the upper excavation free face, rotation of the support structure about the base, and the granular nature of the cohesionless sand. Excavation on the upper right side formed a free face, while the pushing action from the left side caused the support structure to rotate about the lower unexcavated zone. As a result, the deformation in the upper part was much larger than that in the lower part, forming a tensile gap at the wall-soil interface that widened from top to bottom. Because cohesionless sand cannot sustain tensile stress, it collapsed and flowed downward under gravity, causing separation between the upper earth pressure cells and the soil. Consequently, stress could not be effectively transferred to these cells, leading to relatively small earth pressure readings. Therefore, the proposed theoretical model is more applicable to the compacted contact zone in the middle and lower portions of the soil mass, whereas its predictions are conservative in the upper low-stress zone, where wall-soil contact relaxation may occur.

The proposed theoretical model is more applicable to the primary compression zone where effective wall-soil contact is maintained. In the upper region, where pronounced separation, near-zero normal contact stress, or discontinuous contact may occur, the corresponding earth pressure should not be directly included as passive resistance in stability assessments and may be reduced appropriately. In future models, the upper earth pressure may be further corrected by incorporating wall-soil separation or tensile-crack mechanisms and introducing a contact-relaxation condition.

## 4. Parametric analysis

To avoid extending the parametric analysis beyond the experimental validation range, this study examined only the effects of the support inclination angle and soil internal friction angle on the resultant passive earth pressure and the height of its point of application under the condition of B/H = 0.95. The geometric scale and material parameters were consistent with those adopted in the model tests: backfill height *H* = 0.4 m, backfill width *B* = 0.38m, soil density *γ* = 14.5 k N/m^3^, internal friction angle *φ* = 36°, and wall-soil friction angles *δ*_1_ = *δ*_2_ = 22.8°. When evaluating the effect of the internal friction angle, only *φ* was varied, while *δ*_1_ and *δ*_2_ were set as *δ*_1_ = *δ*_2_ = 2*φ*/3, with all other parameters kept unchanged.

**Fig 18:**
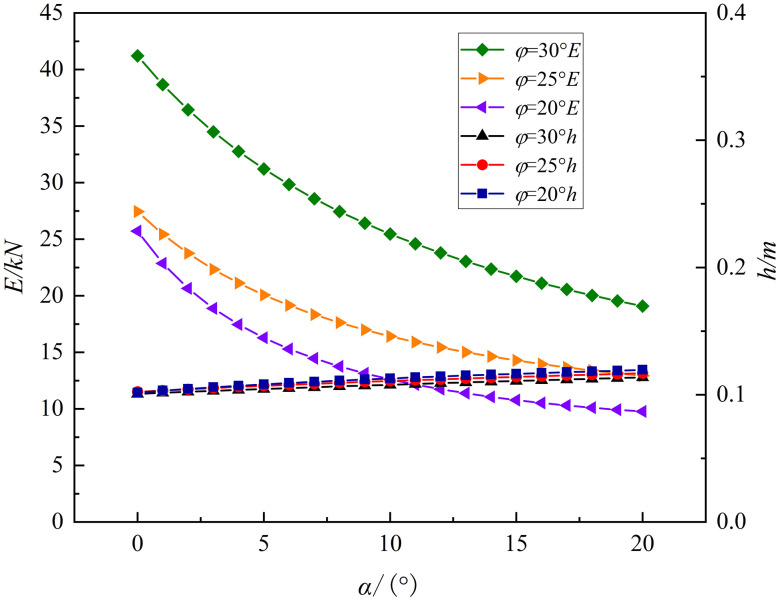
Effect of *φ* on earth pressure.

Fig 18 illustrates the effect of *φ* on earth pressure. The calculation results obtained using the proposed method show that, when the width-to-height ratio is equal to 0.95, the resultant earth pressure *E* decreases with increasing inclination angle, and a larger internal friction angle *φ* leads to a smaller resultant earth pressure. As the inclination angle increases, the position of the point of application of the resultant force increases almost linearly within the range of *H*/4 to *H*/3. However, the increase is relatively small and has a limited influence. Under the same inclination angle, a larger internal friction angle results in a lower position of the resultant force, and this height is lower than that predicted by the classical Coulomb theory This is because an increase in the internal friction angle enhances the shear strength of the sand and the interlocking effect between particles, making it easier for the soil to form a stable stress transfer structure. Consequently, the upper soil is unloaded under relatively small deformation, while the lower soil bears a larger proportion of the lateral pressure, resulting in a downward shift of the point of application of the resultant force.

## 5. Conclusions

This study investigated the earth pressure acting on inclined support structures adjacent to existing structures through a combination of physical model tests and theoretical derivation. The influence of support inclination angle on the failure surface morphology and earth pressure distribution of a finite soil mass was systematically analyzed. A calculation model for earth pressure on inclined support structures in a finite soil mass was established by considering the soil arching effect. The main conclusions are as follows:

(1) Under the combined action of translational pushing of the adjacent existing structure and the right-side inclined support, the failure mode of the finite soil mass exhibited significant boundary-constrained characteristics. At the limit state, an asymmetric polygonal sliding soil wedge controlled by two failure surfaces was formed.
(2) The earth pressure along the height of the inclined support structure showed a distinctly nonlinear distribution, with stress highly concentrated in the lower region of the retaining wall. The maximum peak earth pressure occurred at the bottom of the wall. In contrast, the readings at the upper measuring points were significantly smaller due to the combined effects of the excavation free face, rotation of the support about the base, and the granular nature of the sand. This is consistent with the PIV observations, which showed that the most intense shear deformation occurred at the wall toe.
(3) Based on the assumptions of planar failure surfaces and circular-arc major principal stress trajectories, a four-zone passive earth pressure calculation model was established by considering the support inclination angle, finite soil mass width, and soil arching effect. By solving the force equilibrium equations of horizontal differential elements in each zone, theoretical models for earth pressure intensity, resultant force, and the point of application of the resultant force were obtained. The theoretical results agreed well with the experimental data overall.
(4) Within the experimental validation range of B/H = 0.95. The parametric analysis showed, an increasing the support inclination angle reduced the resultant earth pressure and shifted the point of application of the resultant force upward, whereas increasing the soil friction angle shifted the point of application downward. The calculated positions were all lower than those obtained using the classical Coulomb theory.
