# Supplementary material for: Earth pressure analysis of inclined support structures adjacent to existing structures
Source: PLoS One. 2026 Jul 30;21(7):e0354972. doi: 10.1371/journal.pone.0354972 (PMC13423047; doi:10.1371/journal.pone.0354972)
Supplement: Attachment — Submitted filename: Response to Reviewers.docx [file pone.0354972.s002.docx]

**Original Manuscript Number:** PONE-D-26-25546

**Original Article Title: “Earth Pressure Analysis of Inclined Support Structures Adjacent to Existing Structures**”

Dear Editor,

We would like to express our sincere gratitude to you and the reviewers for the valuable time and constructive comments devoted to our manuscript (Manuscript Number: PONE-D-26-25546). The insightful suggestions have been instrumental in helping us significantly improve the quality of our work.

We have carefully considered all the comments and have revised the manuscript accordingly. Our point-by-point responses to each comment are detailed below.

We believe the manuscript is now strengthened and hope it meets the journal's standards for publication.

Thank you for your consideration.

Best regards,

<Chucai Peng> et al.

**Reviewer#1**

Concern # 1: The PIV results (Figure 4) and earth pressure measurements (Figure 5) are valuable, but the shear strain contour lines do not clearly indicate the failure surface angles or key points (such as intersections). A quantitative comparison between the observed failure surface angles and the assumed straight failure surfaces (*β* and *ψ*) should be added.

**Author response:** We sincerely thank the reviewer for this valuable comment. We agree that the original manuscript did not provide sufficient clarification regarding the locations of the failure surfaces, their intersection point, or their inclination angles in the PIV shear strain contours. In response to the reviewer’s suggestion, Section 2.2 of the revised manuscript has been supplemented with detailed descriptions of the identification of the first failure surface, the second failure surface, their intersection point, and the failure angles *β* and *ψ*. These observations are further correlated with the assumption of straight failure surfaces adopted in the theoretical model.

Specifically, *β* was determined from the average orientation of the primary shear band originating from the heel of the left retaining wall, whereas *ψ* was determined from the actual location of the second failure surface within the finite soil mass. Under the experimental condition of B/H = 0.95 considered in this study, the failure surface angles observed from the PIV results were consistent with the values of *β* and *ψ* adopted in the theoretical calculations. These revisions strengthen the correspondence between the PIV observations and the assumptions used in the theoretical model.

In addition, the first failure surface, the second failure surface, their intersection point, and the corresponding failure angles have been explicitly marked in Figs. 3 and 5 of the revised manuscript.

Concern # 2: The theoretical derivation assumes a straight failure surface and a circular principal stress trajectory. Although the results are generally consistent with experimental results, the authors should explicitly discuss the model's sensitivity to these assumptions and provide error bounds or uncertainty analysis.

**Author response:** We sincerely thank the reviewer for this important comment. We agree that the assumptions of straight failure surfaces and circular-arc major principal stress trajectories are theoretical simplifications, and that their rationale and applicable range should be further clarified in the manuscript.

In Section 3.1 of the revised manuscript, we explicitly state that the first failure surface angle *β*, and the second failure surface angle *ψ*, were determined according to the locations of the shear bands observed in the PIV results. References [31] and [32] derived theoretical models based on straight and circular-arc failure surface assumptions, respectively. A comparison using identical parameters showed that the calculated earth pressures differed by less than 10%. Therefore, to simplify the calculation procedure, a straight failure surface was adopted in the present study.

In addition, Section 3.3 of the revised manuscript has been supplemented with a comparison between the theoretical predictions and experimental measurements using relative deviations.

Concern # 3: Reservoir earth pressure structure is related to reservoir pressure and energy extraction. This needs to be described in the introduction and cited in at least 3 articles to enhance credibility.

**Author response:** We sincerely thank the reviewer for this valuable suggestion. In response to the reviewer’s comment, the Introduction of the revised manuscript has been expanded to include complex engineering scenarios involving reservoir slopes, reservoir development, and energy extraction. The added discussion addresses the effects of reservoir pressure variation, fluid migration, and extraction-induced disturbances on the stress state, deformation response, and lateral pressure distribution of geomaterials.

Specifically, the revised manuscript includes further discussion of the influences of geological conditions, fluid properties, particle migration, and hydrate reservoir exploitation on stress-transfer paths and local stability states within geological media. Three relevant references [26–28] have also been added. These revisions improve the completeness and credibility of the research background.

Concern # 4: The description of the model test setup (Section 2.1) is good, but the authors should report the repeatability of the test (e.g., how many times each tilt angle was repeated) and quantify the measurement uncertainty of the earth pressure sensor and PIV results.

**Author response:** We sincerely thank the reviewer for this valuable suggestion. In response to the reviewer’s comment, the repeatability of the model tests has been clarified in Section 2.1 of the revised manuscript. Each support inclination angle was tested in triplicate, and the reported earth pressure values represent the averages of the repeated tests. After each test, all soil was removed and the model was refilled in layers to minimize the effects of backfill disturbance and variations in boundary contact conditions on the test results.

Concern # 5: In the theoretical derivation, some equations (e.g., (6), (10), (20), (27)) contain potential transcription or symbol errors. The authors should carefully check all equation numbering and variable consistency.

**Author response:** We sincerely thank the reviewer for identifying this issue. We have conducted a comprehensive review of the equation numbering, variable definitions, and notation throughout the manuscript, with particular attention to the derivations surrounding equations. (6), (10), (20), and (27). Potential transcription errors, formatting issues, and inconsistencies in symbol usage have been corrected and standardized in the revised manuscript.

**Reviewer#2**

Concern # 1: The experimental design only adopts an aspect ratio of 0.95, and the parameter analysis figure 17 shows that the earth pressure increases sharply when the aspect ratio is less than 1, but the measured data of other aspect ratios (such as 0.5, 0.8, 1.2, etc.) are not provided for verification. It is suggested to supplement at least 2~3 groups of comparative tests with different aspect ratios, or explicitly limit the conclusion to around B/H=0.95 to avoid excessive extrapolation.

**Author response:** We sincerely thank the reviewer for this important comment. We agree that the parametric analysis of different width-to-height ratios in the original manuscript was not supported by corresponding experimental data and could lead to excessive extrapolation of the conclusions. Because the physical model tests in this study were conducted only for a finite soil mass with B/H = 0.95, the original analysis concerning variations in the width-to-height ratio should not be directly generalized as a universal conclusion.

In response to the reviewer’s suggestion, the applicability of the conclusions has been explicitly limited to conditions with B/H close to 0.95 in the Abstract, the final paragraph of the Introduction, the parametric analysis, and the Conclusions of the revised manuscript. In addition, to avoid extrapolation beyond the available experimental validation, the subsection entitled “Effect of the Width-to-Height Ratio on Earth Pressure” has been removed. The parametric analysis has been revised to discuss only the effects of the support inclination angle and soil internal friction angle on the resultant passive earth pressure and the height of its point of application under the condition of B/H = 0.95.

Concern # 2: How to determine the inclination *ψ* of the second failure surface in the theoretical model has not given any calculation method or empirical value. In this paper, the PIV results in Figure 4 only show the nephogram of shear strain, but do not quantify the relationship between *ψ* and support inclination angle *α* and soil friction angle *φ*. Please add the basis for determining *ψ*, such as the solution formula based on limit equilibrium or minimum energy principle.

**Author response:** We sincerely thank the reviewer for this valuable suggestion. In response to the reviewer’s comment, the basis for determining the inclination angle of the second failure surface *ψ*, has been further clarified in Section 3.1 of the revised manuscript.

In the present study, *ψ* was not introduced as an independent empirical parameter directly related to the support inclination angle *α*, and the soil internal friction angle *φ*. Instead, under fixed geometric conditions of the finite soil mass, *ψ* was determined from the actual development position of the second failure surface identified from the PIV shear strain contours, together with the geometric relationship associated with the width-to-height ratio B/H, of the finite soil mass. Therefore, under the model test condition of B/H = 0.95, *ψ* was mainly governed by the boundary constraint of the finite soil mass and the actual development position of the second failure surface.

To avoid ambiguity, the revised manuscript explicitly states that this determination method is applicable to the finite soil mass condition with B/H close to 0.95 considered in this study. We also agree with the reviewer that, to establish a general model applicable to different combinations of B/H, *α*, and *φ*, future studies should develop a general solution for *ψ* using limit equilibrium methods, the minimum energy principle, or numerical optimization methods.

Concern # 3: The criterion of limit state is not uniform. In this paper, it is defined that "the failure plane is completely penetrated and the earth pressure reaches the peak value", but Figure 5 does not show the earth pressure time-history curve, and the peak time of different measuring points (T4 and T5) may be different, and it is not verified whether the penetration time of the failure plane shown by PIV is consistent with the peak time. Please provide the earth pressure-displacement curve of typical measuring points, and define the unified quantitative standard of limit state (such as the displacement value corresponding to peak pressure).

**Author response:** We sincerely thank the reviewer for identifying this important issue. We agree that the original manuscript did not provide a sufficiently clear basis for determining the limit state, particularly regarding the correspondence between failure surface development observed from the PIV results and the evolution of earth pressure.

In response to the reviewer’s suggestion, a unified limit-state criterion has been incorporated into Section 2.2 of the revised manuscript. In this study, the limit state is defined as the state at which the primary shear bands become essentially continuous and the resultant earth pressure reaches or approaches its peak value. The corresponding wall displacement is adopted as a unified quantitative criterion. This revision provides a clearer basis for selecting PIV images under different support inclination angles and strengthens the correspondence

Concern # 4: The deviation analysis between the theoretical value and the experimental value is not sufficient. In Figure 16, there is a significant deviation between the theoretical value and the measured value at T1~T3 (the measured value is much smaller than the theoretical value), which is attributed to the void in the upper part and the non-cohesive sand, but the theoretical model does not consider the mechanism of soil-wall separation or tensile crack. Please discuss whether the contact relaxation condition should be introduced in the model, or explain under what conditions the upper earth pressure calculation can be ignored.

**Author response:** We sincerely thank the reviewer for this insightful comment. We agree that substantial discrepancies exist between the theoretical predictions and the measured values at the upper monitoring points T1~T3 in Fig. 16, and that the explanation for these discrepancies in the original manuscript was insufficient.

According to the experimental results, the proposed theoretical model agrees well with the measured values at the middle and lower monitoring points, such as T4 and T5, indicating that the model can reasonably capture the earth pressure distribution within the primary compression zone of the finite soil mass. However, in the upper region represented by T1~T3, the theoretical values are markedly higher than the measured values. This discrepancy is mainly associated with the upper excavation free surface, rotation of the support structure about its base, the granular nature of the cohesionless sand, and wall-soil contact relaxation.

Because the earth pressures in the upper region are relatively small, their influence on the overall resultant earth pressure is limited. For example, under the 0° inclination angle, when the earth pressure above T3 was approximately taken as zero to account for wall-soil contact relaxation, the calculated resultant earth pressure decreased by 10.03%. Therefore, considering both computational simplicity and a conservative safety margin, the calculation method adopted in the manuscript was retained. Nevertheless, we fully agree with the reviewer that the introduction of a contact-relaxation condition would improve the model. In future work, wall-soil separation or tensile-crack mechanisms will be incorporated to correct the earth pressure in the upper region by introducing an appropriate contact-relaxation condition.

Concern # 5: The calculation parameters used in parameter analysis are inconsistent with the scale of model test. Parameter analysis adopts engineering scale parameters such as H=5 m and *γ* = 16 kn/m, while the height of model test is only 0.4 m. The internal friction angle of sand is stress-dependent, and whether the φ value (36) obtained from small-scale test can be directly used for large-scale calculation is not discussed. It is suggested to carry out dimensionless analysis or supplement centrifuge test verification.

**Author response:** We sincerely thank the reviewer for this constructive comment. We agree that the engineering-scale parameters adopted in the original parametric analysis were inconsistent with the scale of the physical model tests, which could affect the interpretation and applicability of the parametric results.

In response to the reviewer’s suggestion, the parametric analysis has been revised. In the revised manuscript, the backfill height H, backfill width B, soil density *ρ*, internal friction angle *ϕ*, and wall-soil friction angles *δ* used in the parametric analysis are all consistent with the model test conditions: H = 0.40m, B = 0.38m, *ρ* = 1.45g/cm^3^, *ϕ* = 36°, and *δ*_1_ = *δ*_2_ = 22.8°. When evaluating the effect of the internal friction angle, only *ϕ* was varied, while *δ*_1_ = *δ*_2_ = 2*ϕ*/3 and all other parameters were kept unchanged.

In addition, the analysis has been limited to the condition of B/H = 0.95 to ensure consistency between the parametric analysis and the experimental validation range. We also acknowledge that the internal friction angle of sand may be affected by stress level, relative density, and scale effects. Therefore, caution should be exercised when directly applying parameters obtained from small-scale model tests to engineering-scale calculations. In future work, dimensional analysis, centrifuge model tests, or numerical simulations will be considered to further validate the applicability of the proposed model under different scale conditions.

Concern # 6: The introduction of research background is weak, and there is a lack of systematic combing of key documents in adjacent construction fields. Although the introduction mentions the classical theory, it does not fully comment on the important progress of passive earth pressure on finite soil in recent years. The lack of these documents leads to the lack of sufficient support for the urgency of research and innovation orientation. It is suggested to supplement at least 5~8 core literatures in related fields in recent five years, and clearly point out that the existing models can not consider the coupling effect of support inclination angle and finite width. Refer to the following documents: Microscale damage evolution of high-temperature granite under liquid nitrogen thermal shock based on computed tomography analysis. Mesoscopic damage enhancement in granite under cyclic liquid nitrogen shocks characterized by computed tomography and texture analysis.

**Author response:** We sincerely thank the reviewer for this valuable suggestion. We agree that the original Introduction did not sufficiently review the research progress on adjacent construction, passive earth pressure in finite soil masses, and soil deformation and stress redistribution under complex boundary conditions.

In response to the reviewer’s suggestion, the Introduction has been supplemented and revised. By referring to the recommended literature, eight relevant references [1–5] and [26–28] have been incorporated to further illustrate the influence of internal structural evolution in geomaterials on their macroscopic mechanical responses under complex disturbance conditions. These revisions strengthen the research background and provide a more comprehensive context for the present study.

Concern # 7: Conclusion (4) The height of the action point calculated in this paper is directly compared with the "classical Coulomb theory", but the Coulomb passive earth pressure theory assumes infinite half space and plane failure surface, which is completely different from the limited soil conditions in this paper, and the comparison benchmark is not clear. Please clearly explain the parameters of Coulomb formula used in the comparison (whether the values of wall back inclination angle, soil friction angle and wall-soil friction angle are consistent with this paper), and discuss the real source of the difference (soil arching effect, boundary restriction or different failure surface shapes) to avoid misleading expression.

**Author response:** We sincerely thank the reviewer for identifying this issue. We agree that directly comparing the calculated height of the point of application of the resultant earth pressure with that predicted by the classical Coulomb theory in the original manuscript could be misleading. The classical Coulomb theory for passive earth pressure is generally based on the assumptions of a semi-infinite soil mass and a planar failure surface. In contrast, the present study concerns a finite soil mass adjacent to an existing structure, where the earth pressure distribution is jointly affected by finite-width boundary constraints, the soil arching effect, the support inclination angle, and variations in failure surface morphology. Therefore, the theoretical assumptions and applicable conditions of the two approaches are not fully equivalent.

In response to the reviewer’s suggestion, the direct comparison between the height of the point of application predicted by the present model and that predicted by the classical Coulomb theory has been removed from the Abstract, parametric analysis, and Conclusions of the revised manuscript. The revised conclusions focus on the earth pressure distribution characteristics, the variation of the resultant earth pressure, and the applicable range of the proposed model for finite soil masses, as established from the physical model tests and theoretical calculations. The revised manuscript further emphasizes that the conclusions of this study are applicable to finite soil mass conditions with B/H close to 0.95.

We would like to thank the Editor and all reviewers again for their careful, professional, and constructive comments. The revisions described above have substantially improved the rigor and readability of the manuscript. We sincerely hope that the revised manuscript will meet the requirements of the journal.
