# Supplementary material for: Earth pressure analysis of inclined support structures adjacent to existing structures
Source: PLoS One. 2026 Jul 30;21(7):e0354972. doi: 10.1371/journal.pone.0354972 (PMC13423047; doi:10.1371/journal.pone.0354972)
Supplement: Peer review history

# Peer review: Decision Letter 0

**DOI:** 10.1371/journal.pone.0354972.r001

**Authors:** Qingchao Li

9 Jun 2026

PONE-D-26-25546

Earth Pressure Analysis of Inclined Support Structures Adjacent to Existing Structures

PLOS One

Dear Dr. Wang,

Thank you for submitting your manuscript to PLOS ONE. After careful consideration, we feel that it has merit but does not fully meet PLOS ONE’s publication criteria as it currently stands. Therefore, we invite you to submit a revised version of the manuscript that addresses the points raised during the review process.

Please submit your revised manuscript by Aug 08 2026 11:59PM. If you will need more time than this to complete your revisions, please reply to this message or contact the journal office at plosone@plos.org. When you're ready to submit your revision, log on to https://www.editorialmanager.com/pone/ and select the 'Submissions Needing Revision' folder to locate your manuscript file.

Please include the following items when submitting your revised manuscript:

- A letter that responds to each point raised by the academic editor and reviewer(s). You should upload this letter as a separate file labeled 'Response to Reviewers'.
- A marked-up copy of your manuscript that highlights changes made to the original version. You should upload this as a separate file labeled 'Revised Manuscript with Track Changes'.
- An unmarked version of your revised paper without tracked changes. You should upload this as a separate file labeled 'Manuscript'.

If you would like to make changes to your financial disclosure, please include your updated statement in your cover letter. Guidelines for resubmitting your figure files are available below the reviewer comments at the end of this letter.

If applicable, we recommend that you deposit your laboratory protocols in protocols.io to enhance the reproducibility of your results. Protocols.io assigns your protocol its own identifier (DOI) so that it can be cited independently in the future. For instructions see: https://journals.plos.org/plosone/s/submission-guidelines#loc-laboratory-protocols. Additionally, PLOS ONE offers an option for publishing peer-reviewed Lab Protocol articles, which describe protocols hosted on protocols.io. Read more information on sharing protocols at https://plos.org/protocols?utm_medium=editorial-email&utm_source=authorletters&utm_campaign=protocols.

As the corresponding author, your ORCID iD is verified in the submission system and will appear in the published article. PLOS supports the use of ORCID, and we encourage all coauthors to register for an ORCID iD and use it as well. Please encourage your coauthors to verify their ORCID iD within the submission system before final acceptance, as unverified ORCID iDs will not appear in the published article. *Only*  the individual author can complete the verification step; PLOS staff *cannot*  verify ORCID iDs on behalf of authors.

We look forward to receiving your revised manuscript.

Kind regards,

Qingchao Li (HPU, China)

Academic Editor

PLOS One

Journal Requirements:

When submitting your revision, we need you to address these additional requirements.

1. Please ensure that your manuscript meets PLOS ONE's style requirements, including those for file naming. The PLOS ONE style templates can be found at

https://journals.plos.org/plosone/s/file?id=wjVg/PLOSOne_formatting_sample_main_body.pdf and

https://journals.plos.org/plosone/s/file?id=ba62/PLOSOne_formatting_sample_title_authors_affiliations.pdf

2. Please note that PLOS One has specific guidelines on code sharing for submissions in which author-generated code underpins the findings in the manuscript. In these cases, we expect all author-generated code to be made available without restrictions upon publication of the work. Please review our guidelines at https://journals.plos.org/plosone/s/materials-and-software-sharing#loc-sharing-code and ensure that your code is shared in a way that follows best practice and facilitates reproducibility and reuse.

3. Thank you for stating in your Funding Statement:

“The study was supported by the Outstanding Youth Project of Natural Science Foundation of Hunan Province, China (Grant No. 24B0598).”

Please provide an amended statement that declares *all* the funding or sources of support (whether external or internal to your organization) received during this study, as detailed online in our guide for authors at http://journals.plos.org/plosone/s/submit-now. Please also include the statement “There was no additional external funding received for this study.” in your updated Funding Statement.

Please include your amended Funding Statement within your cover letter. We will change the online submission form on your behalf.

4. Thank you for stating the following financial disclosure:

“The study was supported by the Outstanding Youth Project of Natural Science Foundation of Hunan Province, China (Grant No. 24B0598).”

Please state what role the funders took in the study. If the funders had no role, please state: "The funders had no role in study design, data collection and analysis, decision to publish, or preparation of the manuscript."

If this statement is not correct you must amend it as needed.

Please include this amended Role of Funder statement in your cover letter; we will change the online submission form on your behalf.

5. Please note that your Data Availability Statement is currently missing the repository name, the DOI/accession number of each dataset and a direct link to access each database. If your manuscript is accepted for publication, you will be asked to provide these details on a very short timeline. We therefore suggest that you provide this information now, though we will not hold up the peer review process if you are unable.

6. We note that Figure 3 in your submission contain copyrighted images. All PLOS content is published under the Creative Commons Attribution License (CC BY 4.0), which means that the manuscript, images, and Supporting Information files will be freely available online, and any third party is permitted to access, download, copy, distribute, and use these materials in any way, even commercially, with proper attribution. For more information, see our copyright guidelines: http://journals.plos.org/plosone/s/licenses-and-copyright.

We require you to either (1) present written permission from the copyright holder to publish these figures specifically under the CC BY 4.0 license, or (2) remove the figures from your submission:

a. You may seek permission from the original copyright holder of Figure 3 to publish the content specifically under the CC BY 4.0 license.

We recommend that you contact the original copyright holder with the Content Permission Form (http://journals.plos.org/plosone/s/file?id=7c09/content-permission-form.pdf) and the following text:

“I request permission for the open-access journal PLOS ONE to publish XXX under the Creative Commons Attribution License (CCAL) CC BY 4.0 (http://creativecommons.org/licenses/by/4.0/). Please be aware that this license allows unrestricted use and distribution, even commercially, by third parties. Please reply and provide explicit written permission to publish XXX under a CC BY license and complete the attached form.”

Please upload the completed Content Permission Form or other proof of granted permissions as an "Other" file with your submission.

In the figure caption of the copyrighted figure, please include the following text: “Reprinted from [ref] under a CC BY license, with permission from [name of publisher], original copyright [original copyright year].”

b. If you are unable to obtain permission from the original copyright holder to publish these figures under the CC BY 4.0 license or if the copyright holder’s requirements are incompatible with the CC BY 4.0 license, please either i) remove the figure or ii) supply a replacement figure that complies with the CC BY 4.0 license. Please check copyright information on all replacement figures and update the figure caption with source information. If applicable, please specify in the figure caption text when a figure is similar but not identical to the original image and is therefore for illustrative purposes only.

7. If the reviewer comments include a recommendation to cite specific previously published works, please review and evaluate these publications to determine whether they are relevant and should be cited. There is no requirement to cite these works unless the editor has indicated otherwise.

Additional Editor Comments:

After carefully considering the reviewers’ comments and evaluating the manuscript, I agree that several important issues remain to be addressed before the manuscript can be considered for publication. The reviewers have identified concerns regarding the methodology, data interpretation, and/or presentation of the results that require substantial revision and clarification. Therefore, I recommend a major revision and encourage the authors to provide a detailed point-by-point response to all reviewer comments in the next submission.

[Note: HTML markup is below. Please do not edit.]

Reviewers' comments:

Reviewer's Responses to Questions

**Comments to the Author**

1. Is the manuscript technically sound, and do the data support the conclusions?

The manuscript must describe a technically sound piece of scientific research with data that supports the conclusions. Experiments must have been conducted rigorously, with appropriate controls, replication, and sample sizes. The conclusions must be drawn appropriately based on the data presented.

Reviewer #1: Yes

Reviewer #2: Yes

**********

2. Has the statistical analysis been performed appropriately and rigorously?

Reviewer #1: Yes

Reviewer #2: Yes

**********

3. Have the authors made all data underlying the findings in their manuscript fully available?

The PLOS Data policy requires authors to make all data underlying the findings described in their manuscript fully available without restriction, with rare exception (please refer to the Data Availability Statement in the manuscript PDF file). The data should be provided as part of the manuscript or its supporting information, or deposited to a public repository. For example, in addition to summary statistics, the data points behind means, medians and variance measures should be available. If there are restrictions on publicly sharing data—e.g. participant privacy or use of data from a third party—those must be specified.

Reviewer #1: Yes

Reviewer #2: Yes

**********

4. Is the manuscript presented in an intelligible fashion and written in standard English?

PLOS ONE does not copyedit accepted manuscripts, so the language in submitted articles must be clear, correct, and unambiguous. Any typographical or grammatical errors should be corrected at revision, so please note any specific errors here.

Reviewer #1: Yes

Reviewer #2: Yes

**********

5. Review Comments to the Author

Please use the space provided to explain your answers to the questions above. You may also include additional comments for the author, including concerns about dual publication, research ethics, or publication ethics. (Please upload your review as an attachment if it exceeds 20,000 characters)

Reviewer #1: 1: The PIV results (Figure 4) and earth pressure measurements (Figure 5) are valuable, but the shear strain contour lines do not clearly indicate the failure surface angles or key points (such as intersections). A quantitative comparison between the observed failure surface angles and the assumed straight failure surfaces (β and ψ) should be added.

2: The theoretical derivation assumes a straight failure surface and a circular principal stress trajectory. Although the results are generally consistent with experimental results, the authors should explicitly discuss the model's sensitivity to these assumptions and provide error bounds or uncertainty analysis.

3: Reservoir earth pressure structure is related to reservoir pressure and energy extraction. This needs to be described in the introduction and cited in at least 3 articles to enhance credibility.

4:The description of the model test setup (Section 2.1) is good, but the authors should report the repeatability of the test (e.g., how many times each tilt angle was repeated) and quantify the measurement uncertainty of the earth pressure sensor and PIV results.

5: In the theoretical derivation, some equations (e.g., (6), (10), (20), (27)) contain potential transcription or symbol errors. The authors should carefully check all equation numbering and variable consistency.

Reviewer #2: Aiming at the passive earth pressure problem of inclined supporting structure under the background of adjacent existing structures, combined with physical model test and theoretical derivation, this study reveals the failure surface shape of finite soil and the nonlinear distribution characteristics of earth pressure, which is innovative in considering the coupling effect of supporting inclination angle and finite width. However, the single test condition (only fixed width-height ratio of 0.95), strong theoretical model assumptions and limited verification data still lead to obvious shortcomings in the universality and engineering popularization value of the conclusions.

1. The experimental design only adopts an aspect ratio of 0.95, and the parameter analysis figure 17 shows that the earth pressure increases sharply when the aspect ratio is less than 1, but the measured data of other aspect ratios (such as 0.5, 0.8, 1.2, etc.) are not provided for verification. It is suggested to supplement at least 2~3 groups of comparative tests with different aspect ratios, or explicitly limit the conclusion to around B/H=0.95 to avoid excessive extrapolation.

2. How to determine the inclination ψ of the second failure surface in the theoretical model has not given any calculation method or empirical value. In this paper, the PIV results in Figure 4 only show the nephogram of shear strain, but do not quantify the relationship between ψ and support inclination angle α and soil friction angle ϕ. Please add the basis for determining ψ, such as the solution formula based on limit equilibrium or minimum energy principle.

3. The criterion of limit state is not uniform. In this paper, it is defined that "the failure plane is completely penetrated and the earth pressure reaches the peak value", but Figure 5 does not show the earth pressure time-history curve, and the peak time of different measuring points (T4 and T5) may be different, and it is not verified whether the penetration time of the failure plane shown by PIV is consistent with the peak time. Please provide the earth pressure-displacement curve of typical measuring points, and define the unified quantitative standard of limit state (such as the displacement value corresponding to peak pressure).

4. The deviation analysis between the theoretical value and the experimental value is not sufficient. In Figure 16, there is a significant deviation between the theoretical value and the measured value at T1~T3 (the measured value is much smaller than the theoretical value), which is attributed to the void in the upper part and the non-cohesive sand, but the theoretical model does not consider the mechanism of soil-wall separation or tensile crack. Please discuss whether the contact relaxation condition should be introduced in the model, or explain under what conditions the upper earth pressure calculation can be ignored.

5. The calculation parameters used in parameter analysis are inconsistent with the scale of model test. Parameter analysis adopts engineering scale parameters such as H=5 m and γ = 16 kn/m, while the height of model test is only 0.4 m. The internal friction angle of sand is stress-dependent, and whether the ϕ value (36) obtained from small-scale test can be directly used for large-scale calculation is not discussed. It is suggested to carry out dimensionless analysis or supplement centrifuge test verification.

6. The introduction of research background is weak, and there is a lack of systematic combing of key documents in adjacent construction fields. Although the introduction mentions the classical theory, it does not fully comment on the important progress of passive earth pressure on finite soil in recent years. The lack of these documents leads to the lack of sufficient support for the urgency of research and innovation orientation. It is suggested to supplement at least 5~8 core literatures in related fields in recent five years, and clearly point out that the existing models can not consider the coupling effect of support inclination angle and finite width. Refer to the following documents: Microscale damage evolution of high-temperature granite under liquid nitrogen thermal shock based on computed tomography analysis. Mesoscopic damage enhancement in granite under cyclic liquid nitrogen shocks characterized by computed tomography and texture analysis.

7. Conclusion (4) The height of the action point calculated in this paper is directly compared with the "classical Coulomb theory", but the Coulomb passive earth pressure theory assumes infinite half space and plane failure surface, which is completely different from the limited soil conditions in this paper, and the comparison benchmark is not clear. Please clearly explain the parameters of Coulomb formula used in the comparison (whether the values of wall back inclination angle, soil friction angle and wall-soil friction angle are consistent with this paper), and discuss the real source of the difference (soil arching effect, boundary restriction or different failure surface shapes) to avoid misleading expression.

**********

6. PLOS authors have the option to publish the peer review history of their article (what does this mean?). If published, this will include your full peer review and any attached files.

If you choose “no”, your identity will remain anonymous but your review may still be made public.

**Do you want your identity to be public for this peer review?**  For information about this choice, including consent withdrawal, please see our Privacy Policy.

Reviewer #1: No

Reviewer #2: No

**********

[NOTE: If reviewer comments were submitted as an attachment file, they will be attached to this email and accessible via the submission site. Please log into your account, locate the manuscript record, and check for the action link "View Attachments". If this link does not appear, there are no attachment files.]

To ensure your figures meet our technical requirements, please review our figure guidelines: https://journals.plos.org/plosone/s/figures

You may also use PLOS’s free figure tool, NAAS, to help you prepare publication quality figures: https://journals.plos.org/plosone/s/figures#loc-tools-for-figure-preparation.

NAAS will assess whether your figures meet our technical requirements by comparing each figure against our figure specifications.

# Author comment: Author response to Decision Letter 1

**DOI:** 10.1371/journal.pone.0354972.r002

29 Jun 2026

Reviewer#1

Concern # 1: The PIV results (Figure 4) and earth pressure measurements (Figure 5) are valuable, but the shear strain contour lines do not clearly indicate the failure surface angles or key points (such as intersections). A quantitative comparison between the observed failure surface angles and the assumed straight failure surfaces (β and ψ) should be added.

Author response: We sincerely thank the reviewer for this valuable comment. We agree that the original manuscript did not provide sufficient clarification regarding the locations of the failure surfaces, their intersection point, or their inclination angles in the PIV shear strain contours. In response to the reviewer’s suggestion, Section 2.2 of the revised manuscript has been supplemented with detailed descriptions of the identification of the first failure surface, the second failure surface, their intersection point, and the failure angles β and ψ. These observations are further correlated with the assumption of straight failure surfaces adopted in the theoretical model.

Specifically, β was determined from the average orientation of the primary shear band originating from the heel of the left retaining wall, whereas ψ was determined from the actual location of the second failure surface within the finite soil mass. Under the experimental condition of B/H = 0.95 considered in this study, the failure surface angles observed from the PIV results were consistent with the values of β and ψ adopted in the theoretical calculations. These revisions strengthen the correspondence between the PIV observations and the assumptions used in the theoretical model.

In addition, the first failure surface, the second failure surface, their intersection point, and the corresponding failure angles have been explicitly marked in Figs. 3 and 5 of the revised manuscript.

Concern # 2: The theoretical derivation assumes a straight failure surface and a circular principal stress trajectory. Although the results are generally consistent with experimental results, the authors should explicitly discuss the model's sensitivity to these assumptions and provide error bounds or uncertainty analysis.

Author response: We sincerely thank the reviewer for this important comment. We agree that the assumptions of straight failure surfaces and circular-arc major principal stress trajectories are theoretical simplifications, and that their rationale and applicable range should be further clarified in the manuscript.

In Section 3.1 of the revised manuscript, we explicitly state that the first failure surface angle β, and the second failure surface angle ψ, were determined according to the locations of the shear bands observed in the PIV results. References [31] and [32] derived theoretical models based on straight and circular-arc failure surface assumptions, respectively. A comparison using identical parameters showed that the calculated earth pressures differed by less than 10%. Therefore, to simplify the calculation procedure, a straight failure surface was adopted in the present study.

In addition, Section 3.3 of the revised manuscript has been supplemented with a comparison between the theoretical predictions and experimental measurements using relative deviations.

Concern # 3: Reservoir earth pressure structure is related to reservoir pressure and energy extraction. This needs to be described in the introduction and cited in at least 3 articles to enhance credibility.

Author response: We sincerely thank the reviewer for this valuable suggestion. In response to the reviewer’s comment, the Introduction of the revised manuscript has been expanded to include complex engineering scenarios involving reservoir slopes, reservoir development, and energy extraction. The added discussion addresses the effects of reservoir pressure variation, fluid migration, and extraction-induced disturbances on the stress state, deformation response, and lateral pressure distribution of geomaterials.

Specifically, the revised manuscript includes further discussion of the influences of geological conditions, fluid properties, particle migration, and hydrate reservoir exploitation on stress-transfer paths and local stability states within geological media. Three relevant references [26–28] have also been added. These revisions improve the completeness and credibility of the research background.

Concern # 4: The description of the model test setup (Section 2.1) is good, but the authors should report the repeatability of the test (e.g., how many times each tilt angle was repeated) and quantify the measurement uncertainty of the earth pressure sensor and PIV results.

Author response: We sincerely thank the reviewer for this valuable suggestion. In response to the reviewer’s comment, the repeatability of the model tests has been clarified in Section 2.1 of the revised manuscript. Each support inclination angle was tested in triplicate, and the reported earth pressure values represent the averages of the repeated tests. After each test, all soil was removed and the model was refilled in layers to minimize the effects of backfill disturbance and variations in boundary contact conditions on the test results.

Concern # 5: In the theoretical derivation, some equations (e.g., (6), (10), (20), (27)) contain potential transcription or symbol errors. The authors should carefully check all equation numbering and variable consistency.

Author response: We sincerely thank the reviewer for identifying this issue. We have conducted a comprehensive review of the equation numbering, variable definitions, and notation throughout the manuscript, with particular attention to the derivations surrounding equations. (6), (10), (20), and (27). Potential transcription errors, formatting issues, and inconsistencies in symbol usage have been corrected and standardized in the revised manuscript.

Reviewer#2

Concern # 1: The experimental design only adopts an aspect ratio of 0.95, and the parameter analysis figure 17 shows that the earth pressure increases sharply when the aspect ratio is less than 1, but the measured data of other aspect ratios (such as 0.5, 0.8, 1.2, etc.) are not provided for verification. It is suggested to supplement at least 2~3 groups of comparative tests with different aspect ratios, or explicitly limit the conclusion to around B/H=0.95 to avoid excessive extrapolation.

Author response: We sincerely thank the reviewer for this important comment. We agree that the parametric analysis of different width-to-height ratios in the original manuscript was not supported by corresponding experimental data and could lead to excessive extrapolation of the conclusions. Because the physical model tests in this study were conducted only for a finite soil mass with B/H = 0.95, the original analysis concerning variations in the width-to-height ratio should not be directly generalized as a universal conclusion.

In response to the reviewer’s suggestion, the applicability of the conclusions has been explicitly limited to conditions with B/H close to 0.95 in the Abstract, the final paragraph of the Introduction, the parametric analysis, and the Conclusions of the revised manuscript. In addition, to avoid extrapolation beyond the available experimental validation, the subsection entitled “Effect of the Width-to-Height Ratio on Earth Pressure” has been removed. The parametric analysis has been revised to discuss only the effects of the support inclination angle and soil internal friction angle on the resultant passive earth pressure and the height of its point of application under the condition of B/H = 0.95.

Concern # 2: How to determine the inclination ψ of the second failure surface in the theoretical model has not given any calculation method or empirical value. In this paper, the PIV results in Figure 4 only show the nephogram of shear strain, but do not quantify the relationship between ψ and support inclination angle α and soil friction angle φ. Please add the basis for determining ψ, such as the solution formula based on limit equilibrium or minimum energy principle.

Author response: We sincerely thank the reviewer for this valuable suggestion. In response to the reviewer’s comment, the basis for determining the inclination angle of the second failure surface ψ, has been further clarified in Section 3.1 of the revised manuscript.

In the present study, ψ was not introduced as an independent empirical parameter directly related to the support inclination angle α, and the soil internal friction angle φ. Instead, under fixed geometric conditions of the finite soil mass, ψ was determined from the actual development position of the second failure surface identified from the PIV shear strain contours, together with the geometric relationship associated with the width-to-height ratio B/H, of the finite soil mass. Therefore, under the model test condition of B/H = 0.95, ψ was mainly governed by the boundary constraint of the finite soil mass and the actual development position of the second failure surface.

To avoid ambiguity, the revised manuscript explicitly states that this determination method is applicable to the finite soil mass condition with B/H close to 0.95 considered in this study. We also agree with the reviewer that, to establish a general model applicable to different combinations of B/H, α, and φ, future studies should develop a general solution for ψ using limit equilibrium methods, the minimum energy principle, or numerical optimization methods.

Concern # 3: The criterion of limit state is not uniform. In this paper, it is defined that "the failure plane is completely penetrated and the earth pressure reaches the peak value", but Figure 5 does not show the earth pressure time-history curve, and the peak time of different measuring points (T4 and T5) may be different, and it is not verified whether the penetration time of the failure plane shown by PIV is consistent with the peak time. Please provide the earth pressure-displacement curve of typical measuring points, and define the unified quantitative standard of limit state (such as the displacement value corresponding to peak pressure).

Author response: We sincerely thank the reviewer for identifying this important issue. We agree that the original manuscript did not provide a sufficiently clear basis for determining the limit state, particularly regarding the correspondence between failure surface development observed from the PIV results and the evolution of earth pressure.

In response to the reviewer’s suggestion, a unified limit-state criterion has been incorporated into Section 2.2 of the revised manuscript. In this study, the limit state is defined as the state at which the primary shear bands become essentially continuous and the resultant earth pressure reaches or approaches its peak value. The corresponding wall displacement is adopted as a unified quantitative criterion. This revision provides a clearer basis for selecting PIV images under different support inclination angles and strengthens the correspondence

Concern # 4: The deviation analysis between the theoretical value and the experimental value is not sufficient. In Figure 16, there is a significant deviation between the theoretical value and the measured value at T1~T3 (the measured value is much smaller than the theoretical value), which is attributed to the void in the upper part and the non-cohesive sand, but the theoretical model does not consider the mechanism of soil-wall separation or tensile crack. Please discuss whether the contact relaxation condition should be introduced in the model, or explain under what conditions the upper earth pressure calculation can be ignored.

Author response: We sincerely thank the reviewer for this insightful comment. We agree that substantial discrepancies exist between the theoretical predictions and the measured values at the upper monitoring points T1~T3 in Fig. 16, and that the explanation for these discrepancies in the original manuscript was insufficient.

According to the experimental results, the proposed theoretical model agrees well with the measured values at the middle and lower monitoring points, such as T4 and T5, indicating that the model can reasonably capture the earth pressure distribution within the primary compression zone of the finite soil mass. However, in the upper region represented by T1~T3, the theoretical values are markedly higher than the measured values. This discrepancy is mainly associated with the upper excavation free surface, rotation of the support structure about its base, the granular nature of the cohesionless sand, and wall-soil contact relaxation.

Because the earth pressures in the upper region are relatively small, their influence on the overall resultant earth pressure is limited. For example, under the 0° inclination angle, when the earth pressure above T3 was approximately taken as zero to account for wall-soil contact relaxation, the calculated resultant earth pressure decreased by 10.03%. Therefore, considering both computational simplicity and a conservative safety margin, the calculation method adopted in the manuscript was retained. Nevertheless, we fully agree with the reviewer that the introduction of a contact-relaxation condition would improve the model. In future work, wall-soil separation or tensile-crack mechanisms will be incorporated to correct the earth pressure in the upper region by introducing an appropriate contact-relaxation condition.

Concern # 5: The calculation parameters used in parameter analysis are inconsistent with the scale of model test. Parameter analysis adopts engineering scale parameters such as H=5 m and γ = 16 kn/m, while the height of model test is only 0.4 m. The internal friction angle of sand is stress-dependent, and whether the φ value (36) obtained from small-scale test can be directly used for large-scale calculation is not discussed. It is suggested to carry out dimensionless analysis or supplement centrifuge test verification.

Author response: We sincerely thank the reviewer for this constructive comment. We agree that the engineering-scale parameters adopted in the original parametric analysis were inconsistent with the scale of the physical model tests, which could affect the interpretation and applicability of the parametric results.

In response to the reviewer’s suggestion, the parametric analysis has been revised. In the revised manuscript, the backfill height H, backfill width B, soil density δ, internal friction angle δ, and wall-soil friction angles δ used in the parametric analysis are all consistent with the model test conditions: H = 0.40m, B = 0.38m, δ = 1.45g/cm3, δ = 36°, and δ1 = δ2 = 22.8°. When evaluating the effect of the internal friction angle, only δ was varied, while δ1 = δ2 = 2δ/3 and all other parameters were kept unchanged.

In addition, the analysis has been limited to the condition of B/H = 0.95 to ensure consistency between the parametric analysis and the experimental validation range. We also acknowledge that the internal friction angle of sand may be affected by stress level, relative density, and scale effects. Therefore, caution should be exercised when directly applying parameters obtained from small-scale model tests to engineering-scale calculations. In future work, dimensional analysis, centrifuge model tests, or numerical simulations will be considered to further validate the applicability of the proposed model under different scale conditions.

Concern # 6: The introduction of research background is weak, and there is a lack of systematic combing of key documents in adjacent construction fields. Although the introduction mentions the classical theory, it does not fully comment on the important progress of passive earth pressure on finite soil in recent years. The lack of these documents leads to the lack of sufficient support for the urgency of research and innovation orientation. It is suggested to supplement at least 5~8 core literatures in related fields in recent five years, and clearly point out that the existing models can not consider the coupling effect of support inclination angle and finite width. Refer to the following documents: Microscale damage evolution of high-temperature granite under liquid nitrogen thermal shock based on computed tomography a

Attachment Submitted filename: Response to Reviewers.docx

# Peer review: Decision Letter 1

**DOI:** 10.1371/journal.pone.0354972.r003

**Authors:** Qingchao Li

15 Jul 2026

Earth Pressure Analysis of Inclined Support Structures Adjacent to Existing Structures

PONE-D-26-25546R1

Dear Dr. Wang,

We’re pleased to inform you that your manuscript has been judged scientifically suitable for publication and will be formally accepted for publication once it meets all outstanding technical requirements.

Within one week, you’ll receive an e-mail detailing the required amendments. When these have been addressed, you’ll receive a formal acceptance letter and your manuscript will be scheduled for publication.

An invoice will be generated when your article is formally accepted. Please note, if your institution has a publishing partnership with PLOS and your article meets the relevant criteria, all or part of your publication costs will be covered. Please make sure your user information is up-to-date by logging into Editorial Manager at Editorial Manager® and clicking the ‘Update My Information' link at the top of the page. For questions related to billing, please contact billing support.

If your institution or institutions have a press office, please notify them about your upcoming paper to help maximize its impact. If they’ll be preparing press materials, please inform our press team as soon as possible -- no later than 48 hours after receiving the formal acceptance. Your manuscript will remain under strict press embargo until 2 pm Eastern Time on the date of publication. For more information, please contact onepress@plos.org.

Kind regards,

Qingchao Li (HPU, China)

Academic Editor

PLOS One

Additional Editor Comments (optional):

Based on the evaluations provided by two independent reviewers, all of whom recommend acceptance, and after careful consideration of the revised manuscript, I am pleased to confirm that the paper has met the journal’s publication standards. The authors have adequately addressed all concerns raised during the review process, and the quality of the manuscript has been significantly improved. Therefore, I hereby accept the manuscript for publication in its current form.

Reviewers' comments:

Reviewer's Responses to Questions

**Comments to the Author**

1. If the authors have adequately addressed your comments raised in a previous round of review and you feel that this manuscript is now acceptable for publication, you may indicate that here to bypass the “Comments to the Author” section, enter your conflict of interest statement in the “Confidential to Editor” section, and submit your "Accept" recommendation.

Reviewer #1: All comments have been addressed

Reviewer #2: (No Response)

**********

2. Is the manuscript technically sound, and do the data support the conclusions?

The manuscript must describe a technically sound piece of scientific research with data that supports the conclusions. Experiments must have been conducted rigorously, with appropriate controls, replication, and sample sizes. The conclusions must be drawn appropriately based on the data presented.

Reviewer #1: Yes

Reviewer #2: (No Response)

**********

3. Has the statistical analysis been performed appropriately and rigorously?

Reviewer #1: Yes

Reviewer #2: (No Response)

**********

4. Have the authors made all data underlying the findings in their manuscript fully available?

The PLOS Data policy requires authors to make all data underlying the findings described in their manuscript fully available without restriction, with rare exception (please refer to the Data Availability Statement in the manuscript PDF file). The data should be provided as part of the manuscript or its supporting information, or deposited to a public repository. For example, in addition to summary statistics, the data points behind means, medians and variance measures should be available. If there are restrictions on publicly sharing data—e.g. participant privacy or use of data from a third party—those must be specified.

Reviewer #1: Yes

Reviewer #2: (No Response)

**********

5. Is the manuscript presented in an intelligible fashion and written in standard English?

PLOS ONE does not copyedit accepted manuscripts, so the language in submitted articles must be clear, correct, and unambiguous. Any typographical or grammatical errors should be corrected at revision, so please note any specific errors here.

Reviewer #1: Yes

Reviewer #2: (No Response)

**********

6. Review Comments to the Author

Please use the space provided to explain your answers to the questions above. You may also include additional comments for the author, including concerns about dual publication, research ethics, or publication ethics. (Please upload your review as an attachment if it exceeds 20,000 characters)

Reviewer #1: The author has answered almost all the questions from the reviewers, and I believe this article can be accepted and published.

Reviewer #2: (No Response)

**********

7. PLOS authors have the option to publish the peer review history of their article (what does this mean?). If published, this will include your full peer review and any attached files.

If you choose “no”, your identity will remain anonymous but your review may still be made public.

**Do you want your identity to be public for this peer review?**  For information about this choice, including consent withdrawal, please see our Privacy Policy.

Reviewer #1: No

Reviewer #2: No

**********

# Editor report: Acceptance letter

**DOI:** 10.1371/journal.pone.0354972.r004

**Authors:** Qingchao Li

PONE-D-26-25546R1

PLOS One

Dear Dr. Wang,

I'm pleased to inform you that your manuscript has been deemed suitable for publication in PLOS One. Congratulations! Your manuscript is now being handed over to our production team.

At this stage, our production department will prepare your paper for publication. This includes ensuring the following:

* All references, tables, and figures are properly cited

* All relevant supporting information is included in the manuscript submission,

* There are no issues that prevent the paper from being properly typeset

You will receive further instructions from the production team, including instructions on how to review your proof when it is ready. Please keep in mind that we are working through a large volume of accepted articles, so please give us a few days to review your paper and let you know the next and final steps.

Lastly, if your institution or institutions have a press office, please let them know about your upcoming paper now to help maximize its impact. If they'll be preparing press materials, please inform our press team within the next 48 hours. Your manuscript will remain under strict press embargo until 2 pm Eastern Time on the date of publication. For more information, please contact onepress@plos.org.

You will receive an invoice from PLOS for your publication fee after your manuscript has reached the completed accept phase. If you receive an email requesting payment before acceptance or for any other service, this may be a phishing scheme. Learn how to identify phishing emails and protect your accounts at https://explore.plos.org/phishing.

If we can help with anything else, please email us at customercare@plos.org.

Thank you for submitting your work to PLOS ONE and supporting open access.

Kind regards,

PLOS ONE Editorial Office Staff

on behalf of

Dr. Qingchao Li

Academic Editor

PLOS One
